# Early-life stress elicits peripheral and brain immune activation differently in wild type and 5xFAD mice in a sex-specific manner

**DOI:** 10.1186/s12974-022-02515-w

**Published:** 2022-06-15

**Authors:** S. Bachiller, I. Hidalgo, M. G. Garcia, A. Boza-Serrano, A. Paulus, Q. Denis, C. Haikal, O. Manouchehrian, O. Klementieva, J. Y. Li, C. J. Pronk, G. K. Gouras, T. Deierborg

**Affiliations:** 1 Experimental Neuroinflammation Laboratory, Department of Experimental Medical Science, Lund University, Lund, Sweden; 2 Present Address: Clinical Unit of Infectious Diseases, Microbiology and Preventive Medicine, Institute of Biomedicine of Sevilla (IBiS), Virgen del Rocío University Hospital, CSIC, University of Sevilla, Seville, Spain; 3 Division of Molecular Hematology, Institution of Laboratory Medicine, Lund University, Lund, Sweden; 4 Experimental Dementia Research Unit, Department of Experimental Medical Science, Lund University, Lund, Sweden; 5 Departamento Bioquímica y Biología Molecular, Facultad de Farmacia, Universidad de Sevilla, Seville, Spain; 6 Medical Microspectroscopy, Department of Experimental Medical Science, Lund University, Lund, Sweden; 7 Neural Plasticity and Repair Unit, Wallenberg Neuroscience Center, Department of Experimental Medical Science, Lund University, Lund, Sweden

**Keywords:** Neuroinflammation, Maternal separation, Early-life stress, Alzheimer’s disease, Sex differences, Immunity

## Abstract

**Background:**

The risk of developing Alzheimer’s disease (AD) is modulated by genetic and environmental factors. Early-life stress (ELS) exposure during critical periods of brain development can impact later brain function and health, including increasing the risk of developing AD. Microglial dysfunction and neuroinflammation have been implicated as playing a role in AD pathology and may be modulated by ELS. To complicate matters further, sex-specific effects have been noted in response to ELS and in the incidence and progression of AD.

**Methods:**

Here, we subjected male and female mice with either a wild type or 5xFAD familial AD-model background to maternal separation (MS) from postnatal day 2 to 14 to induce ELS.

**Results:**

We detected hippocampal neuroinflammatory alterations already at postnatal day 15. By 4 months of age, MS mice presented increased immobility time in the forced swim test and a lower discrimination index in the novel object recognition memory test compared to controls. We found altered *Bdnf* and *Arc* expression in the hippocampus and increased microglial activation in the prefrontal cortex due to MS in a sex-dependent manner. In 5xFAD mice specifically, MS exacerbated amyloid-beta deposition, particularly in females. In the periphery, the immune cell population was altered by MS exposure.

**Conclusion:**

Overall, our results demonstrate that MS has both short- and long-term effects on brain regions related to memory and on the inflammatory system, both in the brain and periphery. These ELS-related effects that are detectable even in adulthood may exacerbate pathology and increase the risk of developing AD via sex-specific mechanisms.

**Supplementary Information:**

The online version contains supplementary material available at 10.1186/s12974-022-02515-w.

## Introduction

Alzheimer’s disease (AD) is the most common cause of dementia, affecting six billion people in USA alone [1]. This progressive disease is characterized by gradual memory loss, and the most common form is late-onset AD, which, in most cases, has no clear inheritable cause. Thus, for many, the risk of developing AD is modulated by an amalgamation of genetic and environmental factors, but the exact mechanisms leading to AD remain elusive. One such environmental risk factor is exposure to adversity during childhood, which can contribute to early-life stress (ELS) and alter brain development [2]. The changes due to ELS may lead to long-lasting, harmful effects, including an increased risk of developing AD (reviewed in [2]). In addition, ELS increases the likelihood of developing major depressive disorder (MDD) [3], which in itself is a risk factor for AD along with other stress-related disorders (for reviews, refer to [4, 5]).

Sex-specific effects have been observed both in response to ELS and the development of AD [6]. Of those who have been exposed to ELS, women tend to be more susceptible to developing a stress-related disorder compared to men. Among those with AD, women are also disproportionately affected compared to men [7]. At a cellular level, sex-related differences are also present in microglia, the resident immune cells of the brain. These differences have been noted as differences in the structure, function, and transcriptomic and proteomic profiles of microglia between males and females [8]. This suggests that there are sex-dependent microglial responses in the context of different disorders and diseases. Within the last decade, neuroinflammation and microglial dysfunction have been identified as key drivers of AD [9]. In addition, more recent evidence has shown altered crosstalk between the brain and peripheral immune systems, which may play a role in the development of neurodegenerative diseases [10]. Therefore, inflammatory alterations may serve as the bridge between ELS-related alterations and increased risk of developing AD [11]. Still, the mechanisms linking ELS and AD remain largely unknown.

Here, we modeled ELS in wild-type and 5xFAD AD-model mice by using a protocol for maternal separation (MS) (reviewed in [12]) and looked for MS- and sex-specific alterations, both as separate main effects and as an interaction effect. We find that MS impacts the inflammatory system in the brain and periphery in a sex-specific manner. In the long-term, MS leads to increased depressive-like behavior and impaired novel object recognition memory depending on genotype and sex. In 5xFAD mice, AD pathology is exacerbated by MS differentially based on sex. Altogether, we provide molecular and cellular clues that may help explain the link between ELS and AD risk and highlight the importance of considering sex-specific responses.

## Materials and methods

### Animals

All experiments were performed in accordance with the guidelines on experimental animal research approved by the Malmö-Lund Ethical Committee for Animal Research in Sweden (Dnr. 5.8. 18-01107/2018). The experiments were conducted on age-matched B6SJL 5xFAD Tg6799 transgenic mice and their wild-type (WT) littermates using the following breeding strategy: two WT female mice were paired with one heterozygous 5xFAD male mouse (9–12 weeks old) per cage. Thus, from the same breeding cage, we obtained both WT and 5xFAD heterozygous male and female littermates. Pups were weaned at postnatal day 30 (P30). Age- and sex-matched littermates were group-housed (4–5 animals/cage) in standard cages on a 12 h light/dark cycle, and water, food and nesting material were provided ad libitum.

### Genotyping

Mice were genotyped for WT or 5xFAD status by PCR as previously described [13]. Briefly, DNA from ear punches collected at P30 was extracted using a kit (Extract-N-Amp™, Sigma-Aldrich) and amplified for PCR using the 2× PCR Bio HS Taq Mix Red enzyme (PCR Biosystems) with the following primers (5′–3′): APP forward: AGGACTGACCACTCGACCAG; APP reverse: CGGGGGTCTAGTTCTGCAT; PSN1 forward: AATAGAGAACGGCAGGAGCA; PSN1 reverse: GCCATGAGGGCACTAATCAT; WT APP forward: CTAGGCCACAGAATTGAAAGATCT; WT APP reverse: GTAGGTGGAAATTCTAGCATCATCC.

### Maternal separation

Maternal separation (MS) was performed as recently reported [14]. Briefly, pups were separated from their dams every day from P2 to P14, 3 h per day (09.00 AM–12.00 PM), and placed in a different room to avoid vocalized communication with their dams. Extra nesting material (cotton pieces) was added to keep them warm. After 180 min, pups were returned to their home cage and left undisturbed until the following MS session. Control litters (non-MS) were handled similarly to the MS pups from P2 to P14 without separation from their dams. No differences in body weight were found at the end of the MS manipulation.

### Experimental design

5xFAD and WT littermate mice were randomly assigned to the Non-MS and MS conditions between P2 and P14. At P15, two cohorts of animals (*n* = 5 animals/group) were sacrificed to analyze microglia and cytokine levels. Behavioral characterization started at 4 months old and was performed in five different cohorts (5–13 animals/group). Following three consecutive days of handling, mice were tested for *anxiety-like* behavior in the open field test (*n* = 6–13 animals/group), learning and memory using the novel object recognition memory test (5–7 animals/group) and *depressive-like* behavior in the forced swim test (6–12 animals/group) (see Fig. 2A). Three days after the last behavioral session, a random subset of animals from each group was perfused, and their brains were collected for subsequent molecular analysis (≥ 4 animals/group). Flow cytometry analyses were performed on the spleens from 25 animals included in the behavioral experiments and 42 animals that did not undergo behavioral testing for a minimum of 5 animals/group.

### Behavioral testing

Behavioral tests were performed in specific behavioral rooms during the light phase (09.00 AM–3.00 PM) by the same researcher. First, mice were habituated to the behavioral room for at least 30 min before starting the test. All equipment was thoroughly cleaned with 70% ethanol at the beginning of the session and between trials to remove olfactory cues. The testing and analyses of videotaped sessions were performed by a blinded, experienced researcher.

#### Open field test

To evaluate *anxiety-like* behavior, the open field test was performed as previously described [15] with minor modifications. Mice were placed facing the wall in an arena (55 × 40 × 40 cm) and allowed to freely explore for 5 min. The session was recorded, and the percentage of the time spent in the center was manually scored and represented.

#### Novel object recognition memory test

To test cognition, we performed the novel object recognition (NOR) memory test as described previously [16]. Briefly, mice were placed in an arena located in the behavioral room with dim lighting and background noise. During the training session, mice were allowed to explore two identical objects for 15 min. One-hour post-training short-term memory was tested by returning the mice to the arena with one object from the training session (familiar object) and one novel object for 10 min. The time spent exploring each object was recorded and manually scored to evaluate the relative exploration of the novel object compared with the familiar object and was calculated as the discrimination index (DI = (*t*_novel_ − *t*_familiar_)/(*t*_novel_ + *t*_familiar_)). Only active exploration was considered, defined as direct interaction with the nose towards the object within a 1.5 cm range and/or touching the object with the nose or vibrissae. Sitting on the object or circling around it was not considered exploratory behavior and not included in the analysis.

#### Forced swim test

To test depressive-like behavior, the forced swim test was performed as previously described [17]. Briefly, mice were individually placed in a cylindrical glass tank filled with water at 23 ± 1 ºC. Each mouse was recorded for 6 min and, at the end of the test, was immediately dried and warmed using a dry paper towel before being returned to their home cage. The time spent immobile, defined as the time spent not moving and floating passively, was manually analyzed from video recordings and represented as a percentage encompassing the last 2 min of the test.

### Sample collection and tissue processing

At P15 and 4 months of age, mice were anesthetized using isoflurane (5%) in oxygen (Virbac) and transcardially perfused with 0.9% saline solution.

#### Brain

Brains were hemisected to enable several means of downstream analysis per mouse. One hemisphere of the brain from P15 and 4-month-old mice was transferred to a 4% paraformaldehyde (PFA, Histolab) solution and fixed overnight at 4 °C. The brains were then transferred to a 30% sucrose solution and stored at 4 °C for 48 h. Coronal sections (40 μm) were sliced using a freezing microtome (Leica SM2000DR) and preserved in a cryoprotective solution (30% sucrose [Sigma-Aldrich], 30% ethylene glycol [Sigma-Aldrich], 40% phosphate-buffered saline) at − 20 °C.

With the other hemisphere, hippocampus and prefrontal cortex were isolated and quickly frozen at − 80 °C for further processing.

#### Spleen

Spleens from 4-month-old mice were harvested and manually dissociated into single cell suspensions in FACS buffer (PBS containing 10% FBS and 0.2 mM EDTA). After erythrocyte lysis with ACK (ammonium-chloride-potassium) lysing buffer for 2 min at room temperature (R/T), total cellularity was determined by direct counting using a Sysmex KX-21 N analyzer (Sysmex).

### Immunofluorescence

Brain sections from P15 and 4-month-old mice were labeled as previously described [14]. Briefly, free-floating coronal sections (40 µm) were permeabilized with 1% (v/v) Triton X-100 in PBS (PBS-T 1%, Sigma-Aldrich) for 1 h and then incubated in blocking solution with 5% normal donkey serum (NDS) in PBS-T 1% for Gal-3, 6E10 and Iba1 co-labeling. For Iba1 and CD68 co-labeling, sections were blocked in 3% NDS in PBS-T 0.3% for 1 h. Sections were then incubated overnight at 4ºC with primary antibodies for microglia (Iba1, Wako, 1:500), Gal-3 (R&D, 1:300), CD68 (BioRad, 1:1000) and APP/amyloid-beta (Aβ) (6E10, Covance, 1:500). The following day, the tissue was rinsed for 1 h in PBS-T 0.1%, incubated with the corresponding secondary antibody (1:500, donkey-anti-rabbit 647 for Iba1, donkey-anti-goat 488 for Gal3, donkey-anti-rat conj. Cy2 for CD68 and donkey-anti-mouse 555 for 6E10, Invitrogen) for 1 h and then mounted with Diamond Antifade Mountant (ThermoScientific, Sweden) for visualization. Images of Iba1 labeling of P15 mice and Iba1, Gal3, and 6E10 co-labeling of 4-month-old mice were taken using a Nikon A1RHD laser-scanning confocal microscope using a 20 × air objective (numerical aperture 0.5). CD68-Iba1 co-labeled sections were visualized with a Nikon Eclipse 80i upright microscope using a 20 × objective (numerical aperture 0.75). All acquisition parameters were kept constant for a given experiment and were taken by the same researcher, blinded to the genotype, sex, and ELS exposure. The fluorescently labeled structures were analyzed using Fiji Image J software (W. Rasband, National Institutes of Health). For analysis, image background was first subtracted before a brightness threshold was set for the measurement of each marker. Confocal images were analyzed from a maximum intensity projection. At P15, hippocampal microglia were morphologically classified into three morphological phenotypes: with ramified processes, with stout processes, and round/ameboid like previously reported [18]. At least 2–3 brain sections per animal and brain region were analyzed at the following Bregma coordinates: hippocampus (− 2.0, − 2.5 mm), prefrontal cortex (+ 2.22, + 1.98 mm), and amygdala (− 1.7, − 2.0 mm).

### Congo red staining

To measure plaque burden, coronal brain sections (40 µm) from 4-month-old 5xFAD mice were mounted onto SuperFrost Plus slides (Thermo Scientific) 24 h before staining with Congo red. Sections were then incubated for 3 min in an alcoholic sodium chloride solution (0.03% (w/v) NaCl in 100% ethanol) with 20% Congo red (Sigma Aldrich) before being dehydrated for visualization. Slides were scanned using a 40 × objective in brightfield mode on a digital scanner (Nanozoomer 2H). Densitometric analysis of the labeled plaques was performed using NDP2-viewer software (Hammamatsu, JP) by a researcher blinded to the sex and ELS exposure.

### Flow cytometry

Spleens were collected and homogenized as described in Sect. "Spleen". Subsequently, a fraction of each sample was divided into two different tubes for labeling with specific antibody cocktails: one for identifying mainly lymphoid cells and the other for identifying mostly myeloid cells. Cells were labeled in FACS buffer for 30 min at 4 °C with the corresponding master mix containing the following antibodies: lymphoid master mix containing CD4-APC Cy7 (GK1.5), NK1.1- Biotin (PK136), CD45.2-FITC (104), CD45.1-FITC (A20), Streptavidin-BV605 (Biolegend), CD8a- PeCy5 (53-6.7), CD11b-APC (M1/70), and CD62L-BV421; and Myeloid master mix containing B220- Biotin (RA3-6B2), NK1.1- Biotin (PK136), CD4-Biotin (GK1.5), CD8a-Biotin (53-6.7), CD45.2-FITC (104), CD45.1-FITC (A20), CD11b-APC (M1/70), CD115-BV605 (AF598), Ly6G-APC/Fire750 (1A8), CD11c-BV570 (N418) (Biolegend), Ly6c-PE (AL-21) and streptavidin-PerCP-Cy5.5 (BD bioscience). After labeling and prior to FACS processing, cells were stained using propidium iodide (Life Technologies) diluted 1:1000 in FACS buffer to determine viability. All samples were processed in an LSR Fortessa analyzer (Becton Dickinson), wherein compensation controls with singly labeled anti-rat/anti-hamster or anti-mouse compensation beads (BD Bioscience) were set up prior to the acquisition. Fluorescence minus one (FMO) controls with unfractionated splenocytes were run for proper gating definition. Acquired data were analyzed using FlowJo software (TreeStar). Immunophenotypic descriptions of analyzed populations include the following: inflammatory monocytes CD45 + Lin-(CD4; CD8; Nk1.1; B220) CD11bhiLy6ChiLy6G-/loCD115 + ; T cytotoxic cells CD45 + CD8 + CD4-Nk1.1-CD11b-; dendritic cells CD45 + CD11c + ; eosinophils CD45 + Lin-(CD4; CD8; Nk1.1; B220) CD115-CD11bhiSSChi; neutrophils CD45 + Lin-(CD4; CD8; Nk1.1; B220) CD115- CD11bhiSSCloLy6G + , activated T helper cells CD45 + CD4 + CD8-Nk1.1-CD11b-CD62L- and Activated T cytotoxic cells CD45 + CD8 + CD4-Nk1.1-CD11b-CD62L-.

### RNA extraction and RT-qPCR analysis

Total hippocampal RNA from 4-month-old mice was extracted using TRI-reagent (Sigma-Aldrich) following manufacturer’s instructions. RNA concentrations were measured using a NanoDrop (2000C, Thermo), and 1 μg of total RNA was converted to cDNA using an iScript™ cDNA synthesis kit (BioRad). Real-time RT-qPCR was performed using the following primer sequences (5′–3′): *Bdnf* [19] (forward: GCGGACCCATGGGACTCT; reverse: CTGCTGCTGTAGTGACCGA) and *Arc *[19] (forward: GCTGAGCTCTGCTCTTCTTCA; reverse GGTGAGCTGAAGCCACAAAT). Amplification was done using a CFX96™ Real-Time System-C1000™ Thermal Cycler (BioRad). Relative gene expression was normalized to mRNA levels of the housekeeping gene *Gapdh* (forward: ACCCAGAAGACTGTGGATGG; reverse: ACACATTGGGGGTAGGAACA).

### Sequential protein extractions

Sequential protein extractions of prefrontal cortex (PFC) samples from 4-month-old mice were performed as previously described [20]. Briefly, to obtain the S1 fraction, tissue was homogenized with a dounce homogenizer in PBS (1 ml/ 100 µg of tissue) and ultracentrifuged for 1 h at 40,000 rpm. The supernatant (S1 fraction) was aliquoted and stored at – 80 ºC. The pellet was dissolved in RIPA buffer (Sigma-Aldrich) and centrifuged at 30,000 rpm for 1 h. The resulting supernatant (S2 fraction) was aliquoted and stored at – 80 ºC. S1 and S2 fractions contained soluble amyloid. RIPA and PBS solutions were prepared with a protein inhibitor (Protein Inhibitor Cocktail, ThermoScientific; PhosphoStop, Roche). To obtain the S3 fraction, which contained protofibrils, the pellet obtained in the S2 fraction was re-dissolved in buffered-SDS (2% SDS, 20 mM Tris–HCl, pH 7.4, 140 mM NaCl) and centrifuged at 30,000 rpm for 1 h, and the supernatant (S3 fraction) was stored at – 80 ºC. To obtain the P3 fraction, which included dense plaques, the pellet obtained in the S3 fraction was dissolved in SDS-urea (20 mM Tris–HCl, pH 7.4, 4% SDS and 8 M urea) and stored at – 80 ºC.

### Western blotting

Total protein concentrations were estimated using the BCA assay following the manufacturer’s guidelines (BCA Protein Assay-Kit, ThermoScientific, Sweden). Proteins were separated out using SDS-PAGE with pre-cast gels (4–20%, Bio-Rad) and transferred to nitrocellulose membranes using the TransBlot Turbo system (Bio-Rad). The membranes were blocked in a blocking solution (3% skim milk in PBS) for 1 h R/T and incubated with anti-human amyloid precursor protein (APP)/amyloid-β (6E10, 1:3000, Covance) overnight at 4 ºC. The following day, the membranes were washed three times in PBS-T20 0.25% followed by incubation with the secondary antibody (peroxidase-conjugated anti-immunoglobulin, 1:10,000, DAKO) and a conjugated actin antibody for 2 h R/T. Blots were developed using the ECL Clarity kit (Bio-Rad) and visualized using a ChemiBlot XRS + (Bio-Rad). Bands were analyzed by densitometry using Fiji ImageJ software (W. Rasband, National Institutes of Health) and normalized to actin signal (arbitrary units).

### ELISA plates

Cytokine levels in hippocampal extracts of P15 mice were measured using a Meso Scale Discovery V-Plex Plus Kit (MSD Mesoscale Discovery, USA) with proinflammatory mouse panels (IFNγ, IL-1β, IL-2, IL-4, IL-5, IL-6, IL-10, IL-12p70, TNF-α and CXCL1) following manufacturer’s protocol as done previously [21]. Cytokines below the detection limit were removed from the analysis.

### Statistics

All statistical analyses were performed using GraphPad Prism 8.0 Software for Macintosh (GraphPad Software, San Diego, CA, USA). In graphs, individual dots represent single mice. Two-way ANOVA followed by Tukey’s multiple comparisons test was performed and the main effects due to sex, MS and the interaction effect between those factors were reported. Statistical 2-way ANOVA differences between groups are stated in Additional file 3: Tables S1–S5. Additionally, three-way ANOVA followed by Tukey’s multiple comparisons test was performed and the main effects due to sex, MS, genotype and the interaction effect between those factors are reported in Additional file 4: Tables S1.1–S5.1. Data are presented as mean ± SD. *P* values ≤ 0.05 were considered statistically significant and are indicated in the figure legends.

## Results

### Maternal separation elicits neuroinflammatory alterations in the hippocampus and prefrontal cortex of P15 WT and 5xFAD mice

**Fig. 1:**
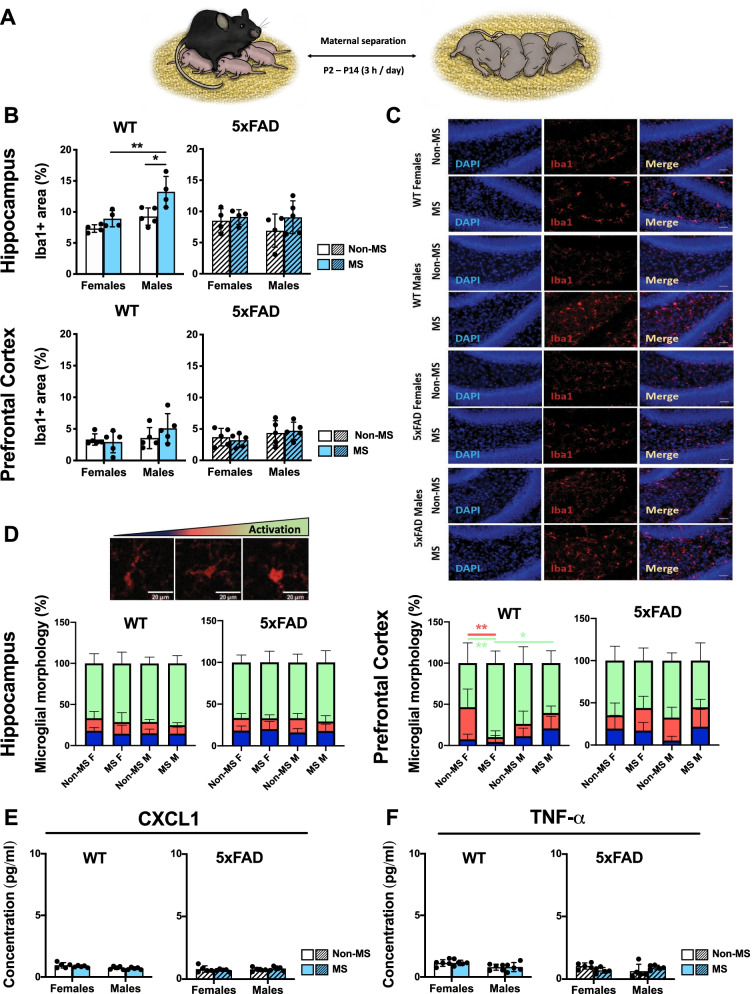
At postnatal day 15 (P15), maternal separation (MS) alters the microglia and the cytokine expression in a sex- and genotype-specific manner. **A** Schematic drawing of the maternal separation protocol. **B** Quantification of the percentage of Iba1-positive area in each group in relation to the total area in hippocampus (**B**, up) and prefrontal cortex (B, down); *n* = 4–5 animals/group; 2–3 sections/animal/area were analyzed. **C** Representative images of Iba1-positive microglia in the hippocampus of each experimental group at P15. **D** Representative microglia activation status and quantification of the percentage of three types of microglia based on their shape: ramified (dark blue bars), with stout processes (red bars) and round/ameboid (light green) in hippocampus (**D**, left) and prefrontal cortex (**D**, right). **E**, **F** Quantification of CXCL1 (**E**) and TNF-a (**F**) in hippocampus using Meso Scale assay; *n* = 5 animals/group with technical duplicates. Scale bar: 20 μm. Data are shown as mean ± SD. *P < 0.05, **P < 0.01

We first wondered whether maternal separation (MS) could induce any immediate neuroinflammatory alterations that we could detect. To study this, we labeled brain tissue from WT and 5xFAD mice 24 h following the last separation session at P15 (Fig. 1A) for the microglial marker Iba1, focusing on the hippocampus (Fig. 1B top and Fig. 1C) and prefrontal cortex (Fig. 1B bottom).

Our results showed significant differences in Iba1 + area between WT groups due to sex (*F*(1,13) = 16.4; *P* = 0.0014) and MS (*F*(1,13) = 13; *P* = 0.0032) in the hippocampus (Fig. 1B top) but not prefrontal cortex (Fig. 1B bottom) (see Additional file 3: Table S1). Similar to a previous study [22], post hoc analysis revealed significantly increased hippocampal Iba1 + area in MS WT male mice compared to their Non-MS counterparts (Fig. 1B) (*P* = 0.0113). Moreover, this increase in microglial area was significantly higher in MS WT males compared to MS WT females (*P* = 0.0093), suggesting a sex-specific microglial response immediately after exposure to MS in WT mice.

In P15 5xFAD mice, we did not find any significant effects on Iba1 + area in the hippocampus nor prefrontal cortex when accounting for sex and MS (see Additional file 3: Table S1). To further characterize microglia at this age, we performed a morphological analysis in both hippocampus and prefrontal cortex and classified microglia as into one of three categories: being ramified, having stout processes or being round/ameboid (Fig. 1D). Despite the differences that we found in Iba1 + area in WT hippocampus, we did not find any differences in the distribution of microglial morphologies between groups in WT and 5xFAD mice (Fig. 1D left, Additional file 3: Table S1). Interestingly, in prefrontal cortex, WT mice showed increased percentages of activated microglia in MS females compared to Non-MS counterparts (with stout processes microglia: *P* = 0.0055; round/ameboid microglia: *P* = 0.0021) and MS males (round/ameboid microglia: *P* = 0.0173) though no differences were found between 5xFAD mouse groups (see Additional file 3: Table S1).

Additionally, we also measured cytokine levels from hippocampal extracts of P15 WT and 5xFAD mice (Fig. 1E–H). In WT mice, a significant sex-dependent difference was observed in CXCL1 (Fig. 1F) and TNF-α (Fig. 1H) levels (CXCL1: *F*(1,16) = 8.93, *P* = 0.0087; TNF-α: *F*(1,16) = 7.36, *P* = 0.0154). In 5xFAD mice, we could only detect a significant interaction effect on TNF-α levels (*F*(1,16) = 4.54, *P* = 0.0489), meaning that the effect of MS differed depending on sex (Fig. 1H; Additional file 3: Table S1). Still, these findings indicate that exposure to MS may alter microglia and cytokine release in a sex-, brain region- and genotype-dependent manner.

### Maternal separation differentially induces depressive-like behavior and cognitive impairment in adult WT and 5xFAD mice

**Fig. 2:**
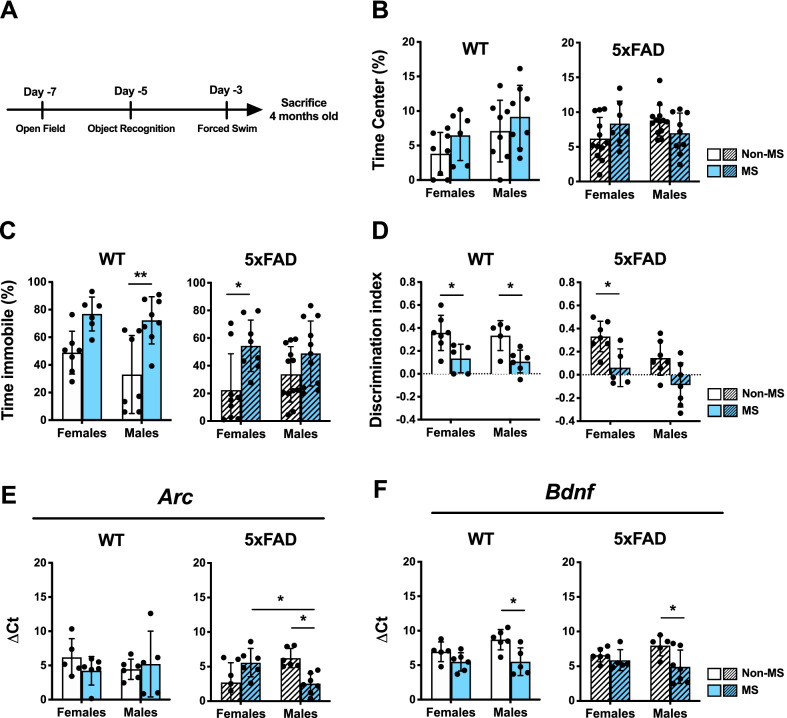
At 4 months, postnatal maternal separation (MS) induces cognitive impairment, and anxiety and depressive-like behavior and alters the hippocampal gene expression in a sex- and genotype-dependent way. **A** Experimental timeline for the behavioral tests at 4 months. **B** *Anxiety-like* behavior was measured as the percentage spent in the center during 5 min in the open field test; *n* = 6–13 animals/group. **C** *Depressive-like* behavior was measured by the percentage spent immobile in the last 2 min of the forced swim test; *n* = 6–12 animals/group. D) Memory performance was evaluated using the novel object recognition memory test; *n* = 5–7 animals/group. Hippocampal gene expression of (**E**) *Bdnf* and (**F**) *Arc* was measured by RT-qPCR and represented as ΔCt value normalized with *Gapdh* gene expression. (*n* = 5-7 animals/group; technical duplicates). Data are shown as mean ± SD. *P < 0.05; **P < 0.01, ***P < 0.001.

We and others have reported sex-dependent behavioral alterations due to MS in adolescent [14] and adult [23] WT mice. Extending this, we wondered whether mice with an AD-like phenotype would demonstrate behavioral alterations due to MS and considered sex in our analyses. Therefore, to evaluate the effects of MS and sex on the behavioral outcomes of adult WT and 5xFAD mice, we performed the open field test (OFT), novel object recognition memory test (NOR) and forced swim test (FST) (Fig. 2A) on 4-month-old mice.

In WT mice, MS significantly affected the behavioral outcomes measured in NOR (Fig. 2D; *F*(1,19) = 17.0; *P* = 0.0006) and FST (Fig. 2C; *F*(1,24) = 20.8, *P* = 0.0001) but not OFT (Fig. 2B). Post hoc analysis revealed that both MS WT females and males presented significantly lower discrimination indices (DI) in NOR compared to their Non-MS counterparts (Fig. 2D). Additionally, MS WT male mice spent more time immobile in FST compared to Non-MS male WT mice (*P* = 0.003), and a trend towards increased immobility time was also observed in MS WT female mice compared to its Non-MS counterpart (Fig. 2C). In WT mice, sex did not have a statistically significant effect on the behavioral outcomes measured (see Additional file 3: Table S2).

In contrast, we observed a significant interaction effect on OFT results in 5xFAD mice (*F*(1,37) = 4.89, *P *= 0.0332; Fig. 2B) with MS having different effects between sexes. We also noted that MS had a significant impact on immobility time in FST (Fig. 2C) and that sex and MS have significant but separate effect on NOR results in 5xFAD mice (Fig. 2D). Specifically, MS 5xFAD females spent more time immobile in FST (*P* = 0.0269) and had a lower DI in NOR (*P* = 0.04) compared to Non-MS 5xFAD females.

### Maternal separation alters hippocampal gene expression in adult mice in a genotype- and sex-specific manner

We next analyzed hippocampal gene expression levels of brain-derived neurotrophic factor (*Bdnf*) and activity-regulated cytoskeleton-associated protein (*Arc*), which are involved in cognition and AD (reviewed in [24, 25]). MS significantly affected the expression of *Bdnf* (Fig. 2F) in both WT (*F*(1,18) = 12.1, *P* = 0.0027) and 5xFAD mice (*F*(1,19) = 6.72, *P *= 0.0179). Post hoc analysis showed significantly lower ΔCt values in both MS WT (*P* = 0.0160) and MS 5xFAD (*P* = 0.034) male mice compared to their Non-MS counterparts though sex did not have an overall statistically significant effect (see Additional file 3: Table S2). Moreover, no differences in the ΔCt values of *Arc* (Fig. 2E) were observed between WT groups (see Additional file 3: Table S2). However, we did find a significant interaction effect in 5xFAD mice on ΔCt values of *Arc* (Interaction: *F*(1,22) = 16.6, *P* = 0.0005; Fig. 2E), indicating a sex-dependent MS effect. Here, post hoc analysis revealed significantly lower ΔCt values in Non-MS females compared to Non-MS males (*P* = 0.0253) and in MS males compared to Non-MS males (*P* = 0.0185). Altogether, these findings suggest that sex may dictate the expression changes of certain hippocampal genes in response to MS and that males may be more affected compared to females.

### Maternal separation alters microglial and amyloid pathology in the prefrontal cortex of 4-month-old 5xFAD mice in a sex-specific pattern

**Fig. 3:**
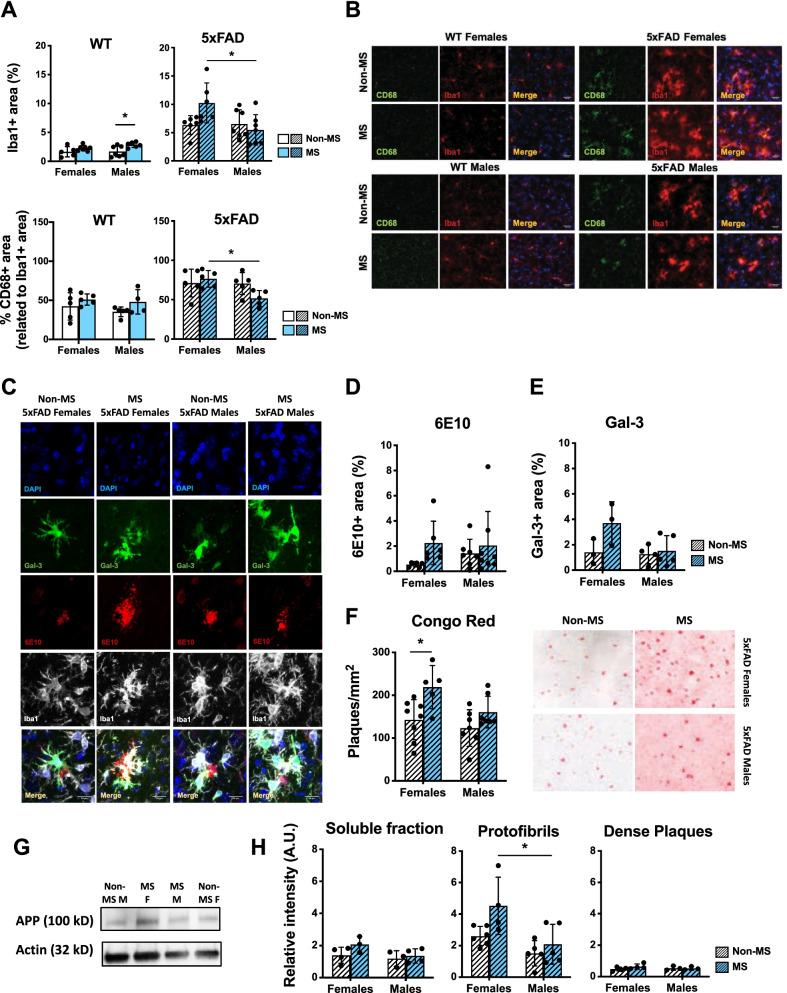
Maternal separation (MS) induces microglial alterations in prefrontal cortex and exacerbates the amyloid pathology in 5xFAD females. (**A**, top) Quantification of the percentage of Iba1 + area relative to the total PFC area and (**A**, bottom) CD68 + area relative to the total Iba1 + area in 2–3 sections/animal; *n* = 4–7 animals/group. **B** Representative microphotographs of microglia in the prefrontal cortex (PFC) of each experimental group at 4 months old using Iba1 and CD68 antibodies. Green: CD68; red: Iba1; blue: DAPI; Scale bar: 20 μm. **C** Representative microphotographs of microglia and Ab plaques in PFC from 5xFAD female and male mice at 4 months old. Microglia were stained using Iba1 (white) and Gal-3 (green) antibodies; Ab plaques were stained using 6E10 antibody (red) and DAPI (blue) for nuclei; scale bar: 20 μm. **D** Quantification of the percentage of 6E10 + area and (**E**) Gal-3 + area in 2–3 sections/animal and relative to the total area of PFC (for 6E10, *n* = 6–8 animals/group; for Gal-3: *n* = 3–5 animals/group). **F** (left) Ab plaque density in PFC of 5xFAD mice was represented (2–3 sections/animal, *n* = 5–8 animals/group). **F** (right) Representative microphotographs of Ab plaques using Congo Red Staining in the PFC of 5xFAD mice. **G** Representative blots of the brain fraction S3 corresponded to protofibrils from PFC of 5xFAD mice. **H** Relative intensity of APP in PFC sequential protein fraction isolation from 4-month-old mice normalized to Actin (soluble fraction: *n* = 3–4 animals/group; protofibrils: *n* = 4–6 animals/group; dense plaques: *n* = 3–4 animals/group; technical duplicates). Data are shown as mean ± SD. *P < 0.05

Because ELS and AD occur at opposite life stages, we wondered whether microglial alterations would persist in adult WT and 5xFAD mice. We observed significant MS-associated microglial alterations in the prefrontal cortex of adult WT mice (*F*(1,21) = 9.61, *P* = 0.0054; Fig. 3A, B). Post hoc analysis revealed higher Iba1 + area in MS male mice compared to Non-MS counterparts (*P* = 0.0365) though the results are not significantly sex-dependent (see Additional file 3: Table S3). In addition, no significant differences were detected between WT groups in the percentage of CD68 + area within Iba1 + area (Fig. 3A, bottom). Moreover, no differences were found in the Iba1 + area in the other regions that we studied, including the hippocampus (dentate gyrus, CA1 and CA3) and amygdala (Additional file 1: Fig. S1A–E, see Additional file 3: Table S5).

Looking at the effect of MS in AD mouse models, it was recently reported that ms can induce the activation of microglia in PFC of *App* knock-in mice [26]. Here, MS alone did not have a statistically significant effect on microglial activation measures in PFC of 5xFAD mice (*F*(1,22) = 1.87, *P* = 0.1850) (Fig. 3A, down and B). However, there was an interaction effect due to MS dependent on sex effect (*F*(1,22) = 5.49, *P* = 0.0286) and a significant difference in general between sexes (*F*(1,22) = 4.77, *P* = 0.04). These effects were similar when comparing the percentages of CD68 + area within Iba1 + area between 5xFAD mouse groups (Sex: (*F*(1,16) = 4.56; *P* = 0.0485). Specifically, MS females had a higher percentage of Iba1 + area and CD68 + /Iba1 + area compared to MS males (*P* = 0.020; *P* = 0.0431, respectively). In addition, a MS-related trend towards increased Gal-3 immunoreactive area (a marker of microglial activation) was observed (Fig. 3C, E; Additional file 3: Table S3).

In AD, amyloid plaques and microglia are often associated. It was previously reported that MS enhanced AD pathology in 9-month-old male APPswe/Ps1dE9 mice [27]. In 5xFAD mice, we only noted a MS-related trend when looking at APP/Aβ immunoreactivity via the antibody 6E10 (Fig. 3D). However, with Congo red staining, we found that plaque burden was significantly different due to sex (*F*(1,23) = 5.11, *P* = 0.0336) and MS (*F*(1,23) = 10.9, *P* = 0.0031) as separate factors in PFC of 4-month-old 5xFAD mice (Fig. 3F). Furthermore, post hoc analysis did not show any MS-dependent differences in males (*P* = 0.4032) but did in females (*P* = 0.0276) (Fig. 3F). In comparison, we detected sex-dependent differences on plaque density in the hippocampus of 5xFAD mice (Additional file 1: Fig. S1F, G) with overall higher plaque density in MS females compared to MS males (*P* = 0.01). No differences were observed in the plaque load in the amygdala (Additional file 1: Fig. S1F–H, see Additional file 3: Table S5).

We further measured the levels of APP in PFC by aggregation state, but found no differences in the levels in the soluble fraction nor dense plaque fraction (Fig. 3G, H; see Additional file 3: Table S3). However, in the fraction corresponding to protofibrils (S3), levels differed significantly due to sex (*F*(1,16) = 11.9, *P* = 0.0033) and MS (*F*(1,16) = 5.87, *P* = 0.0276) as separate factors with post hoc analysis revealing significantly higher levels of APP in MS females compared with MS males (*P* = 0.0256). Altogether, our data suggest that MS could be playing a sex-specific role in worsening Aβ-related pathology in 5xFAD mice with females more adversely affected than males.

### Maternal separation modifies lymphoid and myeloid cell populations in adulthood

**Fig. 4:**
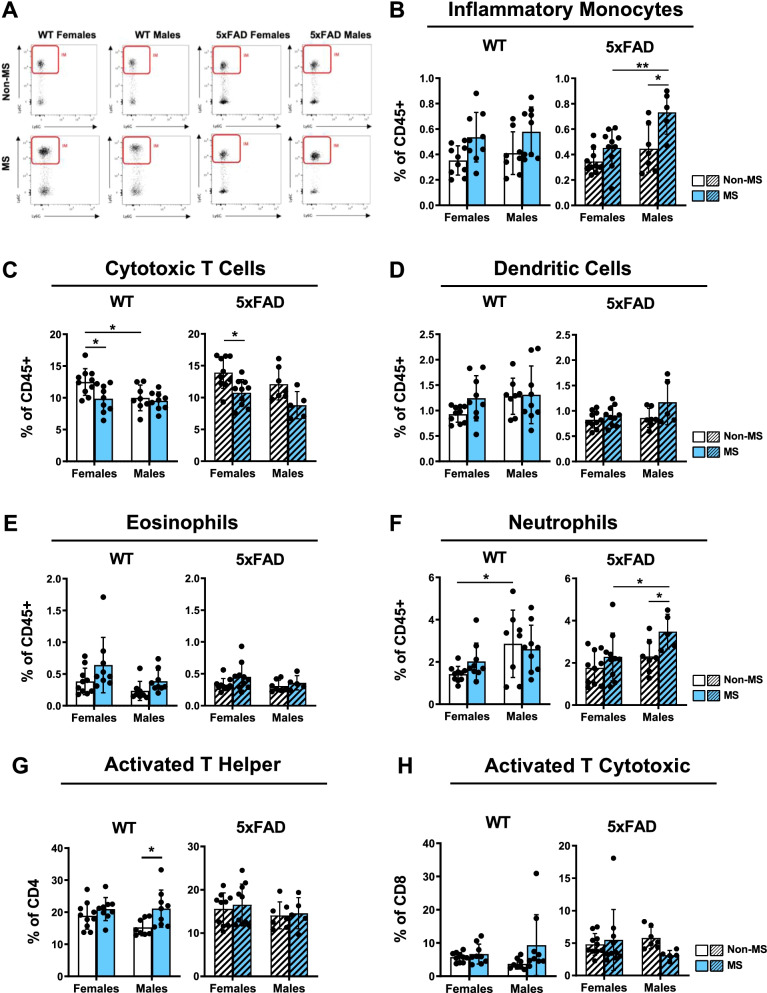
Maternal separation (MS) alters the peripheral immune cells.** A** Contour plots showing inflammatory monocytes gating according to Ly6G and Ly6C expression from cells previously gated on: scatter, singlets; alive; CD45 + Lin- (CD4; CD8; Nk1.1; B220) CD11bhiCD115 + in each experimental group (see Additional file 2: Fig. S2 for complete gating strategy). **B** Analysis of inflammatory monocytes, **C** cytotoxic T cells, **D** dendritic CELLS, **E** Eosinophils, **F** neutrophils, **G** activated T helper and **H** activated T cytotoxic in spleen from WT and 5xFAD mice at 4 months of age (*n* = 5–11 animals/group). Data are shown as mean ± SD. *P < 0.05; **P < 0.01; ***P < 0.001

Though microglia are the resident immune cells in the brain, increasing evidence is showing that microglial crosstalk with peripheral immune cells may be altered in AD and play a role in driving the pathology [10]. As we suspect a link between MS and AD via neuroinflammation, we wanted to assess whether MS could affect the peripheral immune system even into adulthood. To do this, we performed flow cytometry analyses of peripheral inflammatory cells in the spleen, including inflammatory monocytes (Fig. 4A, B), cytotoxic T cells (Fig. 4C), dendritic cells (Fig. 4D), eosinophils (Fig. 4E), neutrophils (Fig. 4F), activated T helper (Fig. 4G) and (Fig. 4H) activated T cytotoxic cells (for gating strategy, see Additional file 2: Fig. S2).

In WT mice, we first observed that the proportion of inflammatory monocytes differed significantly due to MS (*F*(1,32) = 9.61, *P* = 0.004, Fig. 4B) but not sex (see Additional file 3: Table S4). Cytotoxic T cell populations significantly differed based on MS and sex separately (*F*(1,32) = 5.70, *P* = 0.0231; *F*(1,32) = 4.82, *P* = 0.0356, respectively) (Fig. 4C). Non-MS WT females had a proportionally larger population of cytotoxic T cells compared to Non-MS WT males (*P* = 0.0478), and Non-MS WT females had a larger proportion of cytotoxic T cells compared to MS WT females (*P* = 0.0281). No differences in the proportion of dendritic cells (Fig. 4D) were observed between WT groups (see Additional file 3: Table S4). Like the cytotoxic T cell population, ANOVA analysis of eosinophils (Fig. 4E) revealed significant separate effects due to sex (*F*(1,32) = 5.04, *P* = 0.0317) and MS (*F*(1,32) = 5.17, *P* = 0.0298; see Additional file 3: Table S4). Analysis of neutrophils (Fig. 4F) showed a significant difference between sexes (*F*(1,32) = 7.66, *P* = 0.0093) but not MS groups (see Additional file 3: Table S4). Here, post hoc analysis showed that Non-MS males had a significantly larger proportion of neutrophils compared to Non-MS females (*P* = 0.0413). Lastly, the activated T helper (CD4) population (Fig. 4G) was affected by MS (*F*(1,32) = 7.80; *P* = 0.0087) but not sex (see Additional file 3: Table S4). Post hoc analysis revealed a significant increase in this population in MS males compared to Non-MS group (*P* = 0.0379).

In 5xFAD mice, sex (*F*(1,28) = 12.4, *P* = 0.0015) and MS (*F*(1,28) = 13.2, *P* = 0.0011) separately had significant effects on the proportion of inflammatory monocytes. Post hoc analysis revealed a significant increase in the proportion of inflammatory monocytes in MS males compared to Non-MS males (*P* = 0.0123) and MS females (*P* = 0.0085). Likewise, sex (*F*(1,28) = 4.90, *P* = 0.0353) and MS (*F*(1,28) = 14.4, *P* = 0.0007) had separate, significant effects on the proportion of cytotoxic T cells. In this case, Non-MS females had a larger proportion of cytotoxic T cells compared to their MS counterparts (*P* = 0.0227). However, only MS affected the dendritic cell population (*F*(1,28) = 4.74, *P* = 0.0381) in 5xFAD mice, and interestingly, no significant differences were observed in the proportion of eosinophils between groups (see Additional file 3: Table S4). Like inflammatory monocyte and cytotoxic T cell populations, neutrophil populations were also affected by sex (*F*(1,28) = 7.14, *P* = 0.0124) and MS (*F*(1,28) = 5.59, *P* = 0.0253). Lastly, no significant differences were found in the populations of activated T helper (CD4) nor activated T cytotoxic (CD8) cells (Fig. 4H) between the 5xFAD mouse groups (see Additional file 3: Table S4). No interaction effects were detected on any of the cell populations observed in WT and 5xFAD mice. Altogether, these findings suggest that MS can alter the peripheral immune cell profile in the adulthood differently in female and male mice, which may have a detrimental effect in the context of AD.

## Discussion

We sought to elucidate the mechanisms that link ELS to the increased risk of developing AD in vivo, focusing on inflammatory alterations and brain regions particularly affected in AD. To that end, we used MS to induce ELS in WT and 5xFAD AD-model mice. First, we found that at P15, MS alters microglia in a sex-, genotype, and brain region-specific manner as shown by the analysis of Iba1 + area, which increased between WT MS mouse groups only. The microglial morphology in prefrontal cortex was altered with more amoeboid microglia in MS females, but the levels of CXCL1 and TNF-α were unaltered due to MS. Interesting to note, during brain development, microglia density peaks in the mouse hippocampus at P15 [28]. In addition, sex is a crucial factor in microglial maturation as the number of microglial cells begins to differ dramatically between female and male rats from P4 to P30 [18]. We previously characterized the neuroinflammatory profile of untreated 5xFAD mice at timepoints preceding plaque development and reported no significant differences in the cytokine levels of pre-plaque 5xFAD mice compared to age-matched WT male mice [13]. In light of this and the current study, we observed that sex and MS might predispose the brain to be in a reactive, primed state for future challenges. Therefore, though the effects observed at P15 are relatively immediate effects, they likely set the stage for longer-term alterations.

We recently reported that MS altered the neuroinflammatory profile and behavior in adolescent WT mice in a sex-specific manner [14]. In the present study, we furthered this investigation and evaluated neuroinflammatory status and behavioral outcomes due to MS in adult WT and 5xFAD mice and considered sex as a possible factor in our results. Clinical data show that exposure to maternal abuse or neglect is associated with the development of mood disorders and cognitive disturbances in adulthood [3, 29]. These findings have been somewhat recapitulated in rodent models, wherein MS promoted the development of depressive-like behavior [30]. In addition, the development of stress-related disorders is also linked to the risk of developing AD [5] though ELS-induced mood disorders can also be an early manifestation of AD. For example, even before Aβ plaque deposition, depressive-like behavior and memory impairment are already present in male APP/PS1 mice [31]. This sex-specificity is reflected in a study showing that MS induced significantly increased immobility time in the forced swim test in male but not female rats [32]. However, this may need to be reconsidered based on genotype as, in our study, 5xFAD females were the most affected by MS. Regardless, MS overall has been associated with altered behavior, specifically a lower discrimination index in the novel object recognition memory test in rats [33] and other AD mouse models [27]. Although our data suggest that MS may induce behavioral abnormalities in the adulthood regarding depression (as shown by increased immobility time in the forced swim test) and cognition (as shown by decreased discrimination index in the novel object recognition memory test), a larger behavioral phenotyping is needed to further characterize the effect of MS in the adulthood. Nevertheless, these findings are interesting in light of the fact that women are more likely to develop MDD and AD and that ELS may predispose women to both disorders [6, 34].

While behavioral measurements help to serve as a readout for dysfunction at a systems level, behavioral alterations are essentially underlain by changes at the synaptic and cellular levels. Therefore, we assessed hippocampal gene expression of *Arc* and *Bdnf*, which are genes involved in activity-dependent synaptic plasticity, and learning and memory processes [35, 36]. Dysregulation of these genes has been reported in both AD [5] and MDD [37]. Here, we observed decreased *Arc* gene expression together with a lower discrimination index in the novel object recognition memory test in MS 5xFAD male mice. *Arc* expression is regulated by activating glucocorticoid receptors, which are key receptors in the stress response [38]. In aged MS rats [39] and AD-transgenic mice [40], impaired *Arc* expression was associated with deficits in spatial cognition. Moreover, *Arc* has been shown to interact with presenilin-1 and regulate γ-secretase trafficking, which, if altered, may contribute to AD pathogenesis [41]. Regarding *Bdnf* gene expression, similar to previous studies conducted in rodents [42–44], we found genotype-independent significantly lower levels of *Bdnf* transcript in MS males compared to non-MS males. Decreased hippocampal levels of this factor have been associated with AD [45] and depression [46] as well as those with a history of childhood abuse [47].

Another important factor to consider in the ELS response is microglial status. We and others have reported microglial and neuroinflammatory alterations due to MS in adolescent and young adult mice [14, 30]. Sex-differences have been described for female and male microglia that range from cell density and morphology to different functions and regional distribution [48]. For instance, ELS has the ability to activate hippocampal microglia in young male rats [49, 50] but not in females [51], similar to our findings. Additionally, in early developmental stages, similar microglial density is found in cortex independently of sex [8], which could explain the lack of differences in our findings. Moreover, MS has also been shown to induce microglial activation, specifically in male rat pups [49]. These findings led us to consider sex as a crucial factor in response to ELS and its link to AD and associated neuroinflammatory alterations.

Neuroinflammatory processes are important in AD pathogenesis and can be activated by central and peripheral stimuli, such as Aβ plaques and neurofibrillary tangles [52]. In this study, we focused our AD characterization on the PFC area, since we did not find MS-induced differences in microglial activation nor Aβ plaques in hippocampus or amygdala. Specifically, in the 5xFAD MS females, we found increased immunoreactivity of Iba1, Gal-3, and Aβ, which is associated with higher levels of plaque burden and microglial activation, suggesting that ELS experiences could be driving the sex-specific early apparition and/or development of AD in females. However, contrary to what Hui et al. observed [27], we did not see differences between 5xFAD male groups due to MS, which could be explained by the different mouse strain and age that we used. The exposure to ELS during childhood has been associated with a higher incidence of AD and depression [53–55]. Thus, it is tempting to speculate that ELS possibly modulates or “primes” microglia in an early developmental stage. As a result, these cells are unable to respond to pathological Aβ levels in the same way as “non-stressed” microglia, leading to an altered Aβ burden that might be related to the behavioral abnormalities found in this study.

Finally, we evaluated effects on the peripheral immune system by flow cytometry analysis of the spleen, wherein we reported significant sex and MS effects on the myeloid and lymphoid cell populations. Interestingly, genome-wide association studies have pointed out alterations in the transcription factor PU.1, a critical factor for myeloid and B-lymphoid cell development and function as a risk factor for AD [56]. In addition, higher levels of neutrophils and leukocytes have been found in MDD [57], highlighting the importance of peripheral inflammation in mood disorders. Another important factor that may impact microglial activation is the infiltration of immune cells into the brain. In fact, macrophage infiltration is associated with increased inflammation in APP/PS1 AD-model mice [58] and stress-model rodents (reviewed in [59]). Our findings suggest that ELS triggers a long-lasting impact on the immune system and the inflammatory response, which may promote the infiltration of immune cells into the brain. This, in turn, may enhance the neuroinflammatory and microglial responses that could lead to the observed AD phenotype and ELS-induced behavioral disturbances that we observed in MS mice.

## Conclusions

Our study demonstrates that exposure to stress during a sensitive period in early life can modify the inflammatory response in the brain and periphery long-term, potentially leading to behavioral abnormalities in adulthood and enhanced AD pathology in a sex-specific manner.

## Supplementary Information

**Additional file 1: Fig. S1. **Microglia and Ab plaques are not affected by the MS in the hippocampal dentate gyrus, CA1 and CA3 areas and amygdala at 4 months old. (A) Representative microphotographs of microglia (Iba1: white; DAPI: blue) in a whole-brain section of 4 months old mice. Scale bar: 500 μm. Quantification of Iba1 + area relative to the total area in each section from 2–3 sections/animal in (B) dentate gyrus (DG), (C) CA1, (D) CA3 and (E) amygdala (*n* = 5–9 animals/group). (F) Representative Congo Red staining in a whole-brain section of 4 months 5xFAD mice (left) and (right) the total positive plaques/mm2 (*n* = 5–8 animals/group). Data are shown as mean ± SD. *P < 0.05.

**Additional file 2: Fig. S2. **Flow cytometry gating strategy. (A) Lymphoid gating strategy. TOP (from left to right): Exclusion of doublets (FSC-H vs FSC-A), debris (SSC-A vs FSC-A), dead cells (PI +) and selection of white blood cells (CD45 +). MIDDLE (from right to left): Selection of Natural killer (NK) cells, T helper (CD4 +), T cytotoxic (CD8 +) and B lymphocytes (B220 +) according to the sequence marked by the arrows and corresponding markers indicated in Y and X-axis for each plot. BOTTOM: Discrimination of activated T lymphocytes from CD4 or CD8 subsets indicated by arrows and based on the lack of CD62L expression in each case. (B)Myeloid gating strategy. TOP (from left to right): Exclusion of doublets (FSC-H vs FSC-A), debris (SSC-A vs FSC-A), dead cells (PI +) and selection of white blood cells (CD45 +). MIDDLE and BOTTOM: Selection of Dendritic cells (DC), Eosinophils, Neutrophils, Inflammatory Monocytes, and intermediate populations according to the sequence marked by the arrows and corresponding markers indicated in Y and X-axis for each plot.

**Additional file 3:** 2-way ANOVA analysis with Tukey’s multiple comparisons between groups.

**Additional file 4:** 3-way ANOVA analysis with Tukey’s multiple comparisons between groups.

## Data Availability

The data are available from the corresponding author on reasonable request.

## Abbreviations

6E10: Anti-β-amyloid 1–16 (clone 6E10)
AD: Alzheimer’s disease
APP: Amyloid processing protein
*Arc*: Activity-regulated cytoskeleton-associated protein
Aβ: Amyloid-β
*Bdnf*: Brain-derived neurotrophic factor
CA1-3: Cornu ammonis 1–3
DG: Dentate gyrus
DI: Discrimination index
ELS: Early-life stress
FST: Forced swim test
Gal-3: Galectin-3
Iba1: Ionized calcium-binding adaptor molecule 1
MDD: Major depressive disorder
MS: Maternal separation
NOR: Novel object recognition test
OFT: Open field test
P: Postnatal day
PFC: Prefrontal cortex
R/T: Room temperature
WT: Wild type
