# Supplementary figures and images for: Early-life stress elicits peripheral and brain immune activation differently in wild type and 5xFAD mice in a sex-specific manner
Source: J Neuroinflammation. 2022 Jun 15;19:151. doi: 10.1186/s12974-022-02515-w (PMC9199174; doi:10.1186/s12974-022-02515-w)

**A**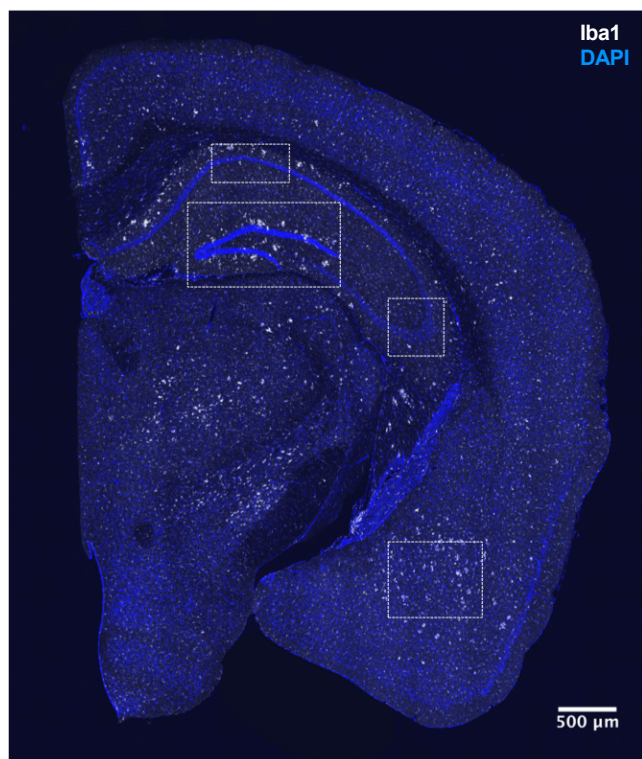**B****Dentate Gyrus**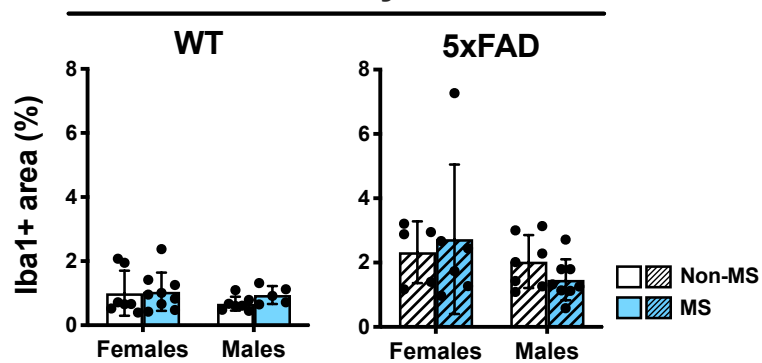**C****CA1**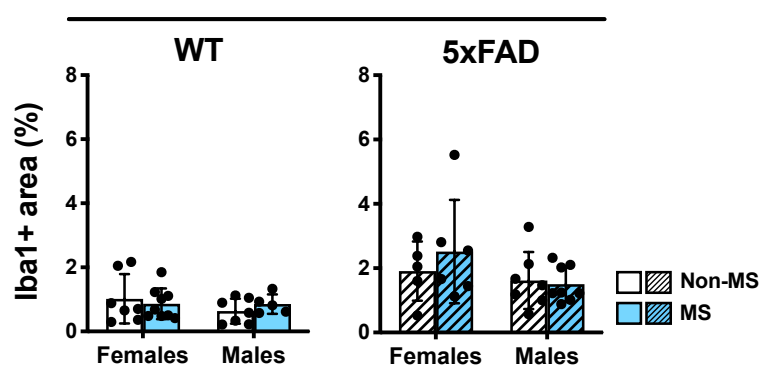**D****CA3**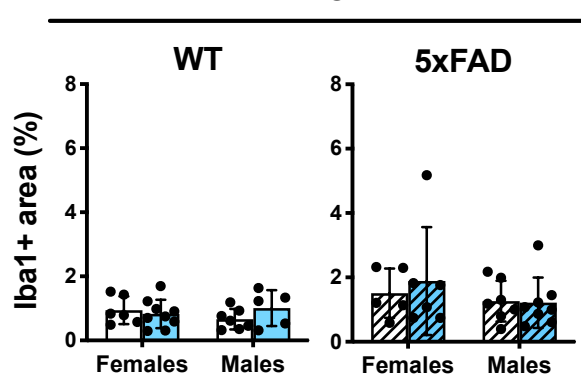**E****Amygdala**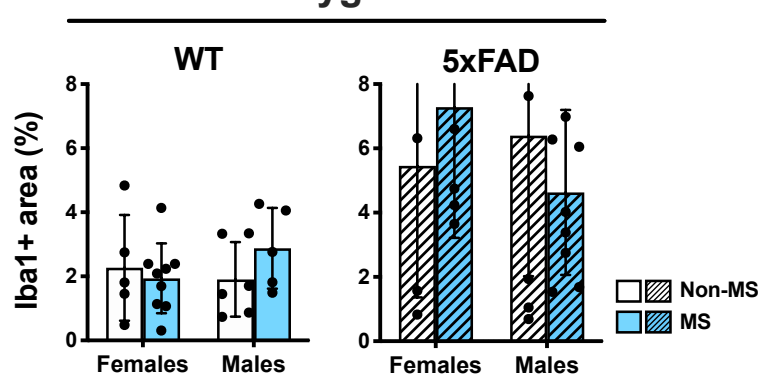**F**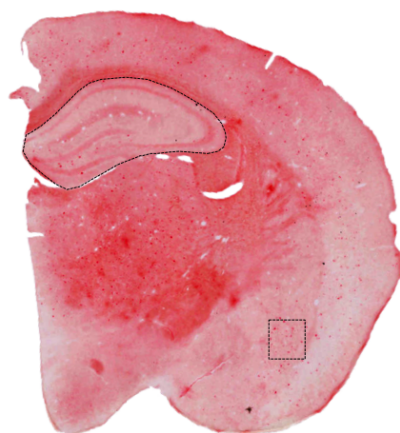**G****Hippocampus**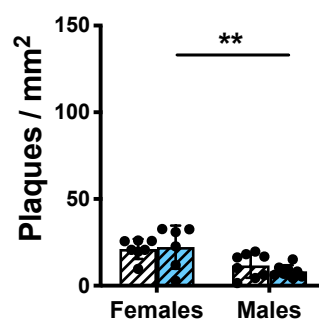**H****Amygdala**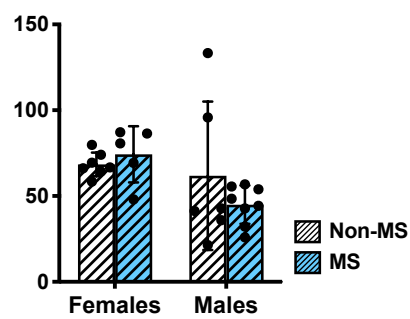

Supplement: Supplementary file 1 — Additional file 1: Fig. S1. Microglia and Ab plaques are not affected by the MS in the hippocampal dentate gyrus, CA1 and CA3 areas and amygdala at 4 months old. (A) Representative microphotographs of microglia (Iba1: white; DAPI: blue) in a whole-brain section of 4 months old mice. Scale bar: 500 μm. Quantification of Iba1 + area relative to the total area in each section from 2–3 sections/animal in (B) dentate gyrus (DG), (C) CA1, (D) CA3 and (E) amygdala (n = 5–9 animals/group). (F) Representative Congo Red staining in a whole-brain section of 4 months 5xFAD mice (left) and (right) the total positive plaques/mm2 (n = 5–8 animals/group). Data are shown as mean ± SD. *P < 0.05. [file 12974_2022_2515_MOESM1_ESM.pdf]

A

# Lymphoid gating strategy

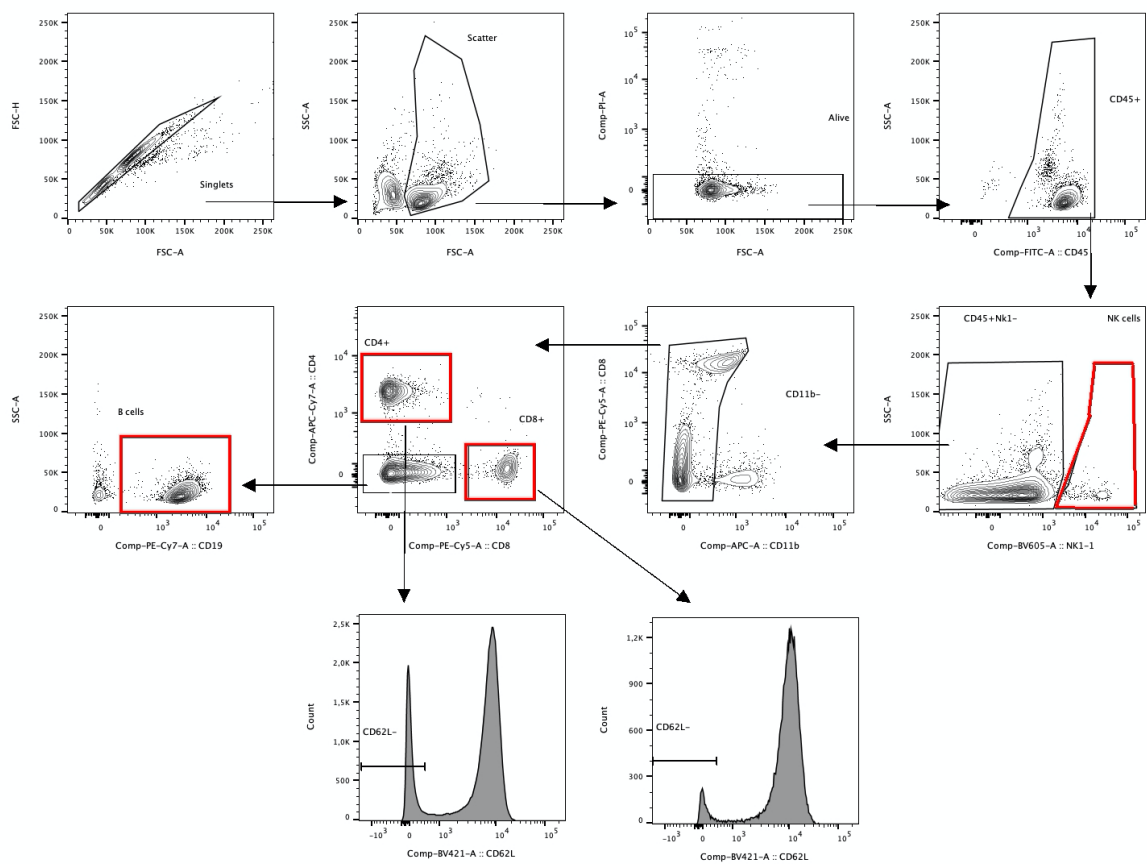

B

# Myeloid gating strategy

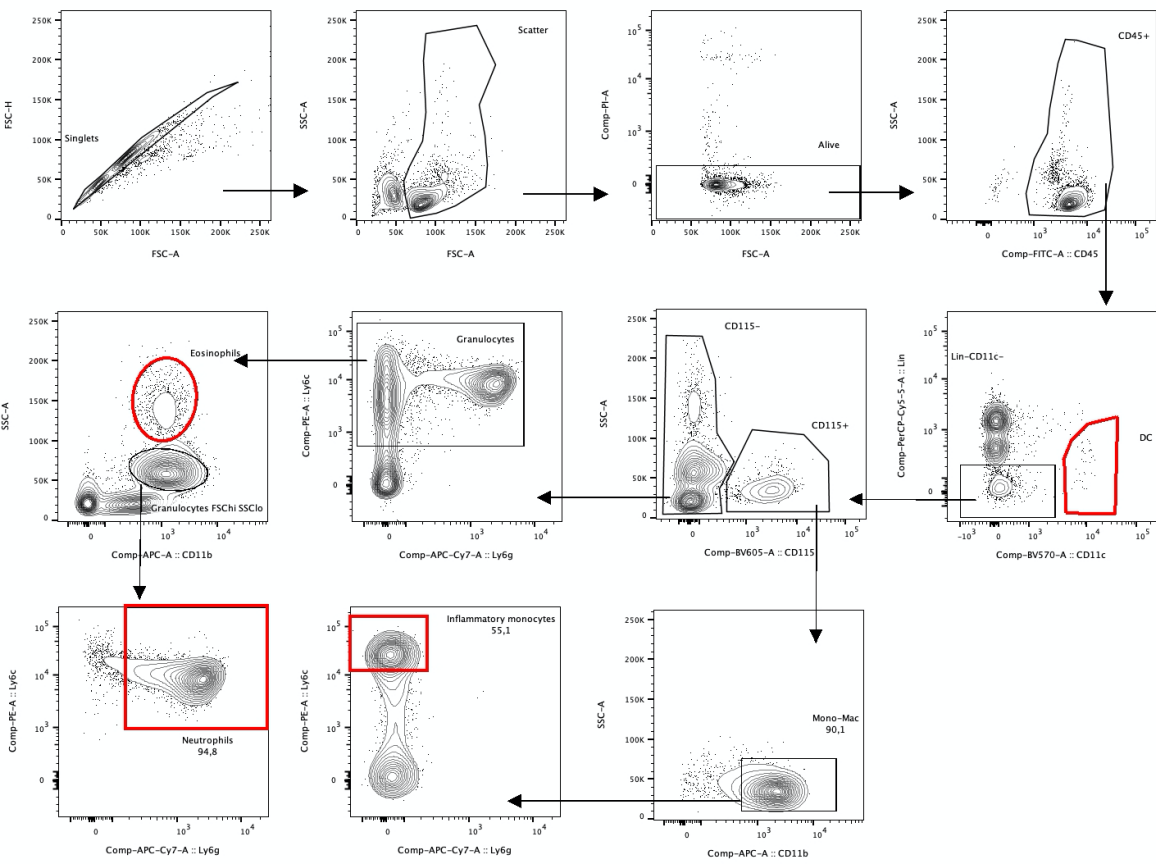

Supplement: Supplementary file 2 — Additional file 2: Fig. S2. Flow cytometry gating strategy. (A) Lymphoid gating strategy. TOP (from left to right): Exclusion of doublets (FSC-H vs FSC-A), debris (SSC-A vs FSC-A), dead cells (PI +) and selection of white blood cells (CD45 +). MIDDLE (from right to left): Selection of Natural killer (NK) cells, T helper (CD4 +), T cytotoxic (CD8 +) and B lymphocytes (B220 +) according to the sequence marked by the arrows and corresponding markers indicated in Y and X-axis for each plot. BOTTOM: Discrimination of activated T lymphocytes from CD4 or CD8 subsets indicated by arrows and based on the lack of CD62L expression in each case. (B)Myeloid gating strategy. TOP (from left to right): Exclusion of doublets (FSC-H vs FSC-A), debris (SSC-A vs FSC-A), dead cells (PI +) and selection of white blood cells (CD45 +). MIDDLE and BOTTOM: Selection of Dendritic cells (DC), Eosinophils, Neutrophils, Inflammatory Monocytes, and intermediate populations according to the sequence marked by the arrows and corresponding markers indicated in Y and X-axis for each plot. [file 12974_2022_2515_MOESM2_ESM.pdf]
