# Supplementary material for: Early-life stress elicits peripheral and brain immune activation differently in wild type and 5xFAD mice in a sex-specific manner
Source: J Neuroinflammation. 2022 Jun 15;19:151. doi: 10.1186/s12974-022-02515-w (PMC9199174; doi:10.1186/s12974-022-02515-w)
Supplement: Supplementary file 3 — Additional file 3: 2-way ANOVA analysis with Tukey’s multiple comparisons between groups. [file 12974_2022_2515_MOESM3_ESM.pdf]

Supplementary Table 1: Statistical analysis for figure 1.

Experiment	Groups	2-way ANOVA			Tukey's multiple comparisons test	
		Factor	F (DFn, DFd)	P Value	Comparisons	P Value
**PD15: Iba1+ area (%) Hippocampus**	**WT**	Interaction<br>**Sex**<br>**MS**	F(1,13)=2.41<br>F(1,13)=16.4<br>F(1,13)=13.0	0.1445<br>**0.0014****<br>**0.0032****	Non-MS F vs. MS F	0.5103
					**Non-MS M vs. MS M**	**0.0113***
					Non-MS F vs. Non-MS M	0.3093
					**MS F vs. MS M**	**0.0093****
	**5xFAD**	Interaction<br>Sex<br>MS	F(1,13)=0.527<br>F(1,13)=0.577<br>F(1,13)=1.610	0.4806<br>0.4610<br>0.2272	Non-MS F vs. MS F	0.9815
					Non-MS M vs. MS M	0.4937
					Non-MS F vs. Non-MS M	0.7388
					MS F vs. MS M	>0.999
**PD15: Iba1+ area (%) Prefrontal Cortex**	**WT**	Interaction<br>Sex<br>MS	F(1,16)=1.59<br>F(1,16)=2.40<br>F(1,16)=0.548	0.2254<br>0.1411<br>0.4699	Non-MS F vs. MS F	0.9823
					Non-MS M vs. MS M	0.5085
					Non-MS F vs. Non-MS M	0.9969
					MS F vs. MS M	0.2337
	**5xFAD**	Interaction<br>Sex<br>MS	F(1,16)=0.420<br>F(1,16)=2.750<br>F(1,16)=0.014	0.5261<br>0.1169<br>0.9055	Non-MS F vs. MS F	0.9469
					Non-MS M vs. MS M	0.9817
					Non-MS F vs. Non-MS M	0.8903
					MS F vs. MS M	0.3906
**PD15: Microglia morphology (%) Hippocampus**	**WT**	Ramified<br>With stout processes<br>Round/ameboid			Non-MS F vs. MS F	0.9380<br>0.9969<br>0.8614
					Non-MS M vs. MS M	0.9999<br>0.9299<br>0.9072
					Non-MS F vs. Non-MS M	0.9529<br>0.9906<br>0.8435
					MS F vs. MS M	>0.9999<br>0.9143

						0.9072
	**5xFAD**	Ramified<br>With stout processes<br>Round/ameboid			Non-MS F vs. MS F	0.9907<br>0.9828<br>0.9999
					Non-MS M vs. MS M	0.9936<br>0.7848<br>0.9057
					Non-MS F vs. Non-MS M	0.9874<br>0.9914<br>>0.9999
					MS F vs. MS M	0.9780<br>0.9947<br>0.9163
**PD15: Microglia morphology (%) Prefrontal Cortex**	**WT**	Ramified<br>With stout processes<br>Round/ameboid			Non-MS F vs. MS F	0.9884<br>**0.0055****<br>**0.0021****
					Non-MS M vs. MS M	0.7510<br>0.9800<br>0.5141
					Non-MS F vs. Non-MS M	0.9780<br>0.0692<br>0.1611
					MS F vs. MS M	0.3246<br>0.5285<br>**0.0173***
	**5xFAD**	Ramified<br>With stout processes<br>Round/ameboid			Non-MS F vs. MS F	0.9941<br>0.6262<br>0.7777
					Non-MS M vs. MS M	0.2831<br>0.9678<br>0.5373
					Non-MS F vs. Non-MS M	0.4133<br>0.6007<br>0.9897
					MS F vs. MS M	0.9573<br>0.9753<br>0.9997
**PD15: CXCL1 Hippocampus**	**WT**	Interaction<br>**Sex**<br>MS	F(1,16)=0.134<br>**F(1,16)=8.93**<br>F(1,16)=1.44	0.7190<br>**0.0087****<br>0.2471	Non-MS F vs. MS F	0.6897
					Non-MS M vs. MS M	0.9335
					Non-MS F vs. Non-MS M	0.1231
					MS F vs. MS M	0.2856
	**5xFAD**	Interaction<br>Sex<br>MS	F(1,15)=1.12<br>F(1,15)=0.441<br>F(1,15)=0.0379	0.3048<br>0.5161<br>0.8481	Non-MS F vs. MS F	0.8114
					Non-MS M vs. MS M	0.9268
					Non-MS F vs. Non-MS M	0.9920

					MS F vs. MS M	0.6241
**PD15: TNF-α Hippocampus**	**WT**	Interaction<br>**Sex**<br>MS	F(1,16)=0.00114<br>**F(1,16)=7.36**<br>F(1,16)=0.00666	0.9735<br>**0.0154***<br>0.9360	Non-MS F vs. MS F	>0.999
					Non-MS M vs. MS M	0.9998
					Non-MS F vs. Non-MS M	0.2502
					MS F vs. MS M	0.2690
	**5xFAD**	**Interaction**<br>Sex<br>MS	**F(1,16)=4.54**<br>F(1,16)=0.374<br>F(1,16)=0.033	**0.0489***<br>0.5494<br>0.8581	Non-MS F vs. MS F	0.3876
					Non-MS M vs. MS M	0.5294
					Non-MS F vs. Non-MS M	0.2511
					MS F vs. MS M	0.7091

4 **Supplementary Table 2:** Statistical analysis for figure 2.

Experiment	Groups	2-way ANOVA			Tukey's multiple comparisons test	
		Factor	F (DFn, DFd)	P Value	Comparisons	P Value
**4 months: Open Field Test**	**WT**	Interaction	F(1,26)=0.0434	0.8366	Non-MS F vs. MS F	0.6126
		Sex	F(1,26)=4.080	0.0537	Non-MS M vs. MS M	0.7368
		MS	F(1,26)=2.560	0.1216	Non-MS F vs. Non-MS M	0.3746
					MS F vs. MS M	0.6110
	**5xFAD**	**Interaction**	F(1,37)=4.890	**0.0332***	Non-MS F vs. MS F	0.3750
		Sex	F(1,37)=0.453	0.5053	Non-MS M vs. MS M	0.4504
		MS	F(1,37)=0.0386	0.8453	Non-MS F vs. Non-MS M	0.1121
					MS F vs. MS M	0.7601
**4 months: Forced Swim Test**	**WT**	Interaction	F(1,24)=0.586	0.4513	Non-MS F vs. MS F	0.0707
		Sex	F(1,24)=1.920	0.1781	**Non-MS M vs. MS M**	**0.003****
		**MS**	F(1,24)=20.80	**0.0001*****	Non-MS F vs. Non-MS M	0.4358
					MS F vs. MS M	0.9714
	**5xFAD**	Interaction	F(1,36)=1.40	0.244	**Non-MS F vs. MS F**	**0.0269***
		Sex	F(1,36)=0.168	0.6845	Non-MS M vs. MS M	0.3792
		**MS**	F(1,36)=10.9	**0.0022****	Non-MS F vs. Non-MS M	0.6567
					MS F vs. MS M	0.9502
**4 months: Novel Object Recognition Memory Test**	**WT**	Interaction	F(1,19)=0.0014	0.9698	**Non-MS F vs. MS F**	**0.0390***
		Sex	F(1,19)=0.213	0.6498	**Non-MS M vs. MS M**	**0.0426***
		**MS**	F(1,19)=17.00	**0.0006*****	Non-MS F vs. Non-MS M	0.9899
					MS F vs. MS M	0.9851
	**5xFAD**	Interaction	F(1,22)=0.0901	0.7668	**Non-MS F vs. MS F**	**0.0400***
		**Sex**	F(1,22)=7.06	**0.0144***	Non-MS M vs. MS M	0.0572
		**MS**	F(1,22)=15.7	**0.0007*****	Non-MS F vs. Non-MS M	0.1563
					MS F vs. MS M	0.4015
**4 months: HP gene expression *Bdnf***	**WT**	Interaction	F(1,18)=1.68	0.2109	Non-MS F vs. MS F	0.4331
		Sex	F(1,18)=1.77	0.1998	**Non-MS M vs. MS M**	**0.0160***
		**MS**	F(1,18)=12.1	**0.0027****	Non-MS F vs. Non-MS M	0.2799
					MS F vs. MS M	>0.9999
	**5xFAD**	Interaction	F(1,19)=2.50	0.1307	Non-MS F vs. MS F	0.8942
		Sex	F(1,19)=0.072	0.7913	**Non-MS M vs. MS M**	**0.034***
		**MS**	F(1,19)=6.72	**0.0179***	Non-MS F vs. Non-MS M	0.5828
					MS F vs. MS M	0.7823
**4 months: HP gene expression *Arc***	**WT**	Interaction	F(1,18)=1.180	0.2918	Non-MS F vs. MS F	0.6909
		Sex	F(1,18)=0.0909	0.7655	Non-MS M vs. MS M	0.9724
		MS	F(1,18)=0.228	0.6391	Non-MS F vs. Non-MS M	0.7618
					MS F vs. MS M	0.9440
	**5xFAD**	**Interaction**	F(1,22)=16.6	**0.0005*****	Non-MS F vs. MS F	0.0842
		Sex	F(1,22)=0.0931	0.7632	**Non-MS M vs. MS M**	**0.0185***
		MS	F(1,22)=0.254	0.6194	**Non-MS F vs. Non-MS M**	**0.0253***

					MS F vs. MS M	0.0634

Supplementary Table 3: Statistical analysis for figure 3.

Experiment	Groups	2-way ANOVA			Tukey's multiple comparisons test	
		Factor	F (DFn, DFd)	P Value	Comparisons	P Value
**4 months: Iba1+ area (%) Prefrontal Cortex**	**WT**	Interaction	F(1,21)=0.898	0.3451	Non-MS F vs. MS F	0.4640
		Sex	F(1,21)=1.26	0.2749	**Non-MS M vs. MS M**	**0.0365***
		**MS**	F(1,21)=9.61	**0.0054****	Non-MS F vs. Non-MS M	0.9994
					MS F vs. MS M	0.4542
	**5xFAD**	**Interaction**	F(1,22)=5.49	**0.0286***	Non-MS F vs. MS F	0.0829
		**Sex**	F(1,22)=4.77	**0.0400***	Non-MS M vs. MS M	0.8893
		MS	F(1,22)=1.87	0.1850	Non-MS F vs. Non-MS M	0.9995
					**MS F vs. MS M**	**0.020***
**4 months: CD68+ area related to Iba1+ area (%) Prefrontal Cortex**	**WT**	Interaction	F(1,15)=0.126	0.7278	Non-MS F vs. MS F	0.6945
		Sex	F(1,15)=0.763	0.3961	Non-MS M vs. MS M	0.4478
		MS	F(1,15)=3.48	0.0819	Non-MS F vs. Non-MS M	0.8074
					MS F vs. MS M	0.9838
	**5xFAD**	Interaction	F(1,16)=4.07	0.0606	Non-MS F vs. MS F	0.9183
		**Sex**	F(1,16)=4.56	**0.0485***	Non-MS M vs. MS M	0.1608
		MS	F(1,16)=1.25	0.2806	Non-MS F vs. Non-MS M	0.9998
					MS F vs. MS M	**0.0431***
**4 months: 6E10+ area (%) Prefrontal Cortex**	**5xFAD**	Interaction	F(1,23)=0.619	0.4396	Non-MS F vs. MS F	0.3647
		Sex	F(1,23)=0.246	0.6246	Non-MS M vs. MS M	0.9044
		MS	F(1,23)=2.86	0.1043	Non-MS F vs. Non-MS M	0.8082
					MS F vs. MS M	0.9967
**4 months: Gal-3+ area (%) Prefrontal Cortex**	**5xFAD**	Interaction	F(1,11)=2.76	0.1251	Non-MS F vs. MS F	0.1313
		Sex	F(1,11)=3.57	0.0853	Non-MS M vs. MS M	0.9875
		MS	F(1,11)=4.30	0.0625	Non-MS F vs. Non-MS M	0.9985
					MS F vs. MS M	0.1029
**4 months: Congo Red Prefrontal Cortex**	**5xFAD**	Interaction	F(1,23)=1.28	0.2967	**Non-MS F vs. MS F**	**0.0276***
		**Sex**	F(1,23)=5.11	**0.0336***	Non-MS M vs. MS M	0.4032
		**MS**	F(1,23)=10.9	**0.0031****	Non-MS F vs. Non-MS M	0.8289
					MS F vs. MS M	0.1365
**4 months: Soluble Fraction Prefrontal Cortex**	**5xFAD**	Interaction	F(1,10)=0.964	0.3494	Non-MS F vs. MS F	0.3138
		Sex	F(1,10)=3.150	0.1061	Non-MS M vs. MS M	0.9690
		MS	F(1,10)=2.60	0.1380	Non-MS F vs. Non-MS M	0.9411
					MS F vs. MS M	0.2684
**4 months: Protofibrils Prefrontal Cortex**	**5xFAD**	Interaction	F(1,16)=1.72	0.2080	Non-MS F vs. MS F	0.0795
		**Sex**	F(1,16)=11.9	**0.0033****	Non-MS M vs. MS M	0.8563
		**MS**	F(1,16)=5.87	**0.0276***	Non-MS F vs. Non-MS M	0.4105
					**MS F vs. MS M**	**0.0256***
**4 months: Dense Plaques Prefrontal Cortex**	**5xFAD**	Interaction	F(1,10)=1.270	0.2867	Non-MS F vs. MS F	0.4491
		Sex	F(1,10)=0.122	0.7343	Non-MS M vs. MS M	0.9987
		MS	F(1,10)=0.804	0.3909	Non-MS F vs. Non-MS M	0.9447
					MS F vs. MS M	0.7296

Supplementary Table 4: Statistical analysis for figure 4.

Experiment	Groups	2-way ANOVA			Tukey's multiple comparisons test	
		**Factor**	**F (DFn, DFd)**	**P Value**	**Comparisons**	**P Value**
**4 months: Inflamm. Monocytes**	**WT**	Interaction	F(1, 32)=0.0145	0.9048	Non-MS F vs. MS F	0.1092
		Sex	F(1, 32)=0.783	0.3827	Non-MS M vs. MS M	0.1913
		**MS**	F(1, 32)=9.61	**0.0040****	Non-MS F vs. Non-MS M	0.8928
					MS F vs. MS M	0.9479
	**5xFAD**	Interaction	F(1, 28)=2.75	0.1086	Non-MS F vs. MS F	0.3789
		**Sex**	F(1, 28)=12.4	**0.0015****	**Non-MS M vs. MS M**	**0.0123***
		**MS**	F(1, 28)=13.2	**0.0011****	Non-MS F vs. Non-MS M	0.5146
					**MS F vs. MS M**	**0.0085***
**4 months: Cytotoxic T Cells**	**WT**	Interaction	F(1, 32)=2.81	0.1033	**Non-MS F vs. MS F**	**0.0281***
		**Sex**	F(1, 32)=4.82	**0.0356***	Non-MS M vs. MS M	0.9611
		**MS**	F(1, 32)=5.70	**0.0231***	**Non-MS F vs. Non-MS M**	**0.0478***
					MS F vs. MS M	0.9828
	**5xFAD**	Interaction	F(1, 28)=0.0043	0.9478	**Non-MS F vs. MS F**	**0.0227***
		**Sex**	F(1, 28)=4.90	**0.0353***	Non-MS M vs. MS M	0.0940
		**MS**	F(1, 28)=14.4	**0.0007*****	Non-MS F vs. Non-MS M	0.3924
					MS F vs. MS M	0.4323
**4 months: Dendritic Cells**	**WT**	Interaction	F(1, 32)=1.10	0.3020	Non-MS F vs. MS F	0.3498
		Sex	F(1, 32)=2.40	0.1312	Non-MS M vs. MS M	0.9989
		MS	F(1, 32)=1.59	0.2163	Non-MS F vs. Non-MS M	0.2777
					MS F vs. MS M	0.9844
	**5xFAD**	Interaction	F(1, 28)=1.76	0.1957	Non-MS F vs. MS F	0.8957
		Sex	F(1, 28)=2.95	0.0971	Non-MS M vs. MS M	0.1470
		**MS**	F(1, 28)=4.74	**0.0381***	Non-MS F vs. Non-MS M	0.9911
					MS F vs. MS M	0.1958
**4 months: Eosinophils**	**WT**	Interaction	F(1, 32)=0.343	0.5625	Non-MS F vs. MS F	0.1810
		**Sex**	F(1, 32)=5.04	**0.0317***	Non-MS M vs. MS M	0.6544
		**MS**	F(1, 32)=5.17	**0.0298***	Non-MS F vs. Non-MS M	0.6493
					MS F vs. MS M	0.2062
	**5xFAD**	Interaction	F(1, 28)=0.482	0.4932	Non-MS F vs. MS F	0.2393
		Sex	F(1, 28)=1.26	0.2712	Non-MS M vs. MS M	0.9314
		MS	F(1, 28)=2.71	0.1107	Non-MS F vs. Non-MS M	0.9884
					MS F vs. MS M	0.6184
**4 months: Neutrophils**	**WT**	Interaction	F(1, 32)=1.43	0.2407	Non-MS F vs. MS F	0.6177
		**Sex**	F(1, 32)=7.66	**0.0093****	Non-MS M vs. MS M	0.9608
		MS	F(1, 32)=0.234	0.6318	**Non-MS F vs. Non-MS M**	**0.0413***
					MS F vs. MS M	0.6831
	**5xFAD**	Interaction	F(1, 28)=0.980	0.3308	Non-MS F vs. MS F	0.6735
		**Sex**	F(1, 28)=7.14	**0.0124***	Non-MS M vs. MS M	0.1747
		**MS**	F(1, 28)=5.59	**0.0253***	Non-MS F vs. Non-MS M	0.5962

					MS F vs. MS M	0.0882
**4 months: Activated T helper**	**WT**	Interaction<br>Sex<br>**MS**	F(1, 32)=1.75<br>F(1, 32)=1.51<br>F(1, 32)=7.80	0.1959<br>0.2287<br>0.0087**	Non-MS F vs. MS F	0.7094
					**Non-MS M vs. MS M**	**0.0379***
					Non-MS F vs. Non-MS M	0.2947
					MS F vs. MS M	0.9999
	**5xFAD**	Interaction<br>Sex<br>MS	F(1, 28)=0.0238<br>F(1, 28)=1.42<br>F(1, 28)=0.24	0.8785<br>0.2441<br>0.6277	Non-MS F vs. MS F	0.9508
					Non-MS M vs. MS M	0.9966
					Non-MS F vs. Non-MS M	0.8654
					MS F vs. MS M	0.8028
**4 months: Activated T cytotoxic**	**WT**	Interaction<br>Sex<br>MS	F(1, 32)=2.02<br>F(1, 32)=0.0467<br>F(1, 32)=4.02	0.1650<br>0.8302<br>0.0536	Non-MS F vs. MS F	0.9739
					Non-MS M vs. MS M	0.1064
					Non-MS F vs. Non-MS M	0.8305
					MS F vs. MS M	0.6551
	**5xFAD**	Interaction<br>Sex<br>MS	F(1, 28)=2.34<br>F(1, 28)=0.398<br>F(1, 28)=0.835	0.1370<br>0.5332<br>0.3687	Non-MS F vs. MS F	0.9564
					Non-MS M vs. MS M	0.4293
					Non-MS F vs. Non-MS M	0.9065
					MS F vs. MS M	0.4777

Supplementary Table 5: Statistical analysis for figure Supplementary 1.

Experiment	Groups	2-way ANOVA			Tukey's multiple comparisons test	
		Factor	F (DFn, DFd)	P Value	Comparisons	P Value
**4 months: Iba1 Dentate Gyrus**	**WT**	Interaction	F(1,24)=0.315	0.5800	Non-MS F vs. MS F	0.9977
		Sex	F(1,24)=1.140	0.2965	Non-MS M vs. MS M	0.8048
		MS	F(1,24)=0.646	0.4296	Non-MS F vs. Non-MS M	0.6469
					MS F vs. MS M	0.9848
	**5xFAD**	Interaction	F(1,22)=0.868	0.3617	Non-MS F vs. MS F	0.9557
		Sex	F(1,22)=2.22	0.1501	Non-MS M vs. MS M	0.8366
		MS	F(1,22)=0.0241	0.8780	Non-MS F vs. Non-MS M	0.9807
					MS F vs. MS M	0.3060
**4 months: Iba1 CA1**	**WT**	Interaction	F(1,24)=0.849	0.3660	Non-MS F vs. MS F	0.9360
		Sex	F(1,24)=0.934	0.3433	Non-MS M vs. MS M	0.8909
		MS	F(1,24)=0.025	0.8744	Non-MS F vs. Non-MS M	0.5328
					MS F vs. MS M	>0.999
	**5xFAD**	Interaction	F(1,22)=0.775	0.3882	Non-MS F vs. MS F	0.7631
		Sex	F(1,22)=2.560	0.1241	Non-MS M vs. MS M	0.9966
		MS	F(1,22)=0.368	0.5501	Non-MS F vs. Non-MS M	0.9608
					MS F vs. MS M	0.2873
**4 months: Iba1 CA3**	**WT**	Interaction	F(1,23)=1.78	0.1950	Non-MS F vs. MS F	0.9610
		Sex	F(1,23)=0.078	0.7826	Non-MS M vs. MS M	0.5378
		MS	F(1,23)=0.465	0.5021	Non-MS F vs. Non-MS M	0.6675
					MS F vs. MS M	0.8776
	**5xFAD**	Interaction	F(1,22)=0.274	0.6061	Non-MS F vs. MS F	0.9294
		Sex	F(1,22)=1.270	0.2716	Non-MS M vs. MS M	0.9997
		MS	F(1,22)=0.157	0.6953	Non-MS F vs. Non-MS M	0.9759
					MS F vs. MS M	0.6227
**4 months: Iba1 Amygdala**	**WT**	Interaction	F(1,21)=1.560	0.2252	Non-MS F vs. MS F	0.9659
		Sex	F(1,21)=0.307	0.5856	Non-MS M vs. MS M	0.5906
		MS	F(1,21)=0.384	0.5419	Non-MS F vs. Non-MS M	0.9642
					MS F vs. MS M	0.5544
	**5xFAD**	Interaction	F(1,24)=1.53	0.2281	Non-MS F vs. MS F	0.8512
		Sex	F(1,24)=0.35	0.5595	Non-MS M vs. MS M	0.7662
		MS	F(1,24)=0.0004	0.9845	Non-MS F vs. Non-MS M	0.9710
					MS F vs. MS M	0.5433
**4 months: Congo Red HP**	**5xFAD**	Interaction	F(1,25)=0.627	0.4359	Non-MS F vs. MS F	0.9936
		**Sex**	F(1,25)=17.6	**0.0003*****	Non-MS M vs. MS M	0.8083
		MS	F(1,25)=0.162	0.6906	Non-MS F vs. Non-MS M	0.0913
					**MS F vs. MS M**	**0.0100**
**4 months: Congo Red Amygdala**	**5xFAD**	Interaction	F(1,22)=1.54	0.2283	Non-MS F vs. MS F	0.9714
		Sex	F(1,22)=3.87	0.0620	Non-MS M vs. MS M	0.5402
		MS	F(1,22)=0.356	0.5570	Non-MS F vs. Non-MS M	0.9533

					MS F vs. MS M	0.1438
