# Supplementary material for: Early-life stress elicits peripheral and brain immune activation differently in wild type and 5xFAD mice in a sex-specific manner
Source: J Neuroinflammation. 2022 Jun 15;19:151. doi: 10.1186/s12974-022-02515-w (PMC9199174; doi:10.1186/s12974-022-02515-w)
Supplement: Supplementary file 4 — Additional file 4: 3-way ANOVA analysis with Tukey’s multiple comparisons between groups. [file 12974_2022_2515_MOESM4_ESM.pdf]

Supplementary Table 1.1: 3-way ANOVA analysis for figure 1.

Experiment	3-way ANOVA			Significant Tukey's multiple comparisons test	
	Factor	F (DFn, DFd)	P Value	Comparisons	P Value
**PD15:<br>Iba1+ area (%)<br>Hippocampus**	1: Sex<br>2: Genotype<br>3: MS<br>4: Sex x (genotype)<br>5: Sex x (MS)<br>6: Genotype x MS<br>7: Sex x (Genotype) x (MS)	1: F(1,26)=3.026<br>2: F(1,26)=3.738<br>3: F(1,26)=9.807<br>4: F(1,26)=8.852<br>5: F(1,26)=2.232<br>6: F(1,26)=1.144<br>7: F(1,26)=0.098	1: 0.938<br>**2: 0.064**<br>**3: 0.004**<br>**4: 0.006**<br>5: 0.147<br>6: 0.294<br>7: 0.7571	WT F Non-MS vs. WT M MS<br>5xFAD F Non-MS vs. WT M MS<br>WT M MS vs. 5xFAD M Non-MS	0.004<br>0.033<br>0.001
**PD15: Iba1+ area (%) Prefrontal Cortex**	1: Sex<br>2: Genotype<br>3: MS<br>4: Sex x (genotype)<br>5: Sex x (MS)<br>6: Genotype x MS<br>7: Sex x (Genotype) x (MS)	1: F(1,32)=5.080<br>2: F(1,32)=0.302<br>3: F(1,32)=0.233<br>4: F(1,32)=0.009<br>5: F(1,32)=1.903<br>6: F(1,32)=10.41<br>7: F(1,32)=0.287	**1: 0.031**<br>2: 0.586<br>3: 0.631<br>4: 0.922<br>5: 0.177<br>6: 0.526<br>7: 0.595	-	
**PD15: Microglia morphology Ramified (%) Hippocampus**	1: Sex<br>2: Genotype<br>3: MS<br>4: Sex x (genotype)<br>5: Sex x (MS)<br>6: Genotype x MS<br>7: Sex x (Genotype) x (MS)	1: F(1,26)=0.546<br>2: F(1,26)=1.003<br>3: F(1,26)=0.002<br>4: F(1,26)=0.024<br>5: F(1,26)=0.079<br>6: F(1,26)=0.540<br>7: F(1,26)=0.118	1: 0.466<br>2: 0.325<br>3: 0.964<br>4: 0.876<br>5: 0.780<br>6: 0.468<br>7: 0.733	-	
**PD15: Microglia morphology With stout processes (%) Hippocampus**	1: Sex<br>2: Genotype<br>3: MS<br>4: Sex x (genotype)<br>5: Sex x (MS)<br>6: Genotype x MS<br>7: Sex x (Genotype) x (MS)	1: F(1,26)=0.335<br>2: F(1,26)=0.120<br>3: F(1,26)=1.892<br>4: F(1,26)=0.430<br>5: F(1,26)=0.361<br>6: F(1,26)=0.120<br>7: F(1,26)=0.013	1: 0.567<br>2: 0.730<br>3: 0.180<br>4: 0.517<br>5: 0.553<br>6: 0.731<br>7: 0.909	-	
**PD15: Microglia morphology Round/Ameboid (%) Hippocampus**	1: Sex<br>2: Genotype<br>3: MS<br>4: Sex x (genotype)<br>5: Sex x (MS)<br>6: Genotype x MS<br>7: Sex x (Genotype) x (MS)	1: F(1,26)=0.640<br>2: F(1,26)=0.691<br>3: F(1,26)=0.680<br>4: F(1,26)=0.078<br>5: F(1,26)=0.029<br>6: F(1,26)=0.068<br>7: F(1,26)=0.080	1: 0.430<br>2: 0.413<br>3: 0.416<br>4: 0.780<br>5: 0.867<br>6: 0.795<br>7: 0.779	-	
**PD15: Microglia morphology: Ramified (%) Prefrontal Cortex**	1: Sex<br>2: Genotype<br>3: MS<br>4: Sex x (genotype)<br>5: Sex x (MS)<br>6: Genotype x MS<br>7: Sex x (Genotype) x (MS)	1: F(1,32)=0.547<br>2: F(1,32)=1.758<br>3: F(1,32)=1.911<br>4: F(1,32)=3.555<br>5: F(1,32)=4.332<br>6: F(1,32)=0.296<br>7: F(1,32)=0.201	1: 0.464<br>2: 0.194<br>3: 0.176<br>4: 0.068<br>**5: 0.045**<br>6: 0.590<br>7: 0.656	-	
**PD15: Microglia morphology with stout processes (%) Prefrontal Cortex**	1: Sex<br>2: Genotype<br>3: MS<br>4: Sex x (genotype)<br>5: Sex x (MS)<br>6: Genotype x MS<br>7: Sex x (Genotype) x (MS)	1: F(1,32)=0.027<br>2: F(1,32)=0.746<br>3: F(1,32)=1.642<br>4: F(1,32)=1.225<br>5: F(1,32)=1.681<br>6: F(1,32)=4.561<br>7: F(1,32)=8.837	1: 0.868<br>2: 0.394<br>3: 0.209<br>4: 0.276<br>5: 0.204<br>**6: 0.040**<br>**7: 0.005**	WT F Non-MS vs. WT F MS	0.011
**PD15: Microglia morphology:**	1: Sex<br>2: Genotype<br>3: MS	1: F(1,32)=0.103<br>2: F(1,32)=2.339<br>3: F(1,32)=0.010	1: 0.749<br>2: 0.136<br>3: 0.919	WT F Non-MS vs. WT F MS	0.046

**Round/Ameboid (%) Prefrontal Cortex**	4: Sex x (genotype)<br>5: Sex x (MS)<br>6: Genotype x MS<br>7: Sex x (Genotype) x (MS)	4: F(1,32)=0.251<br>5: F(1,32)=5.706<br>6: F(1,32)=3.918<br>7: F(1,32)=4.262	4: 0.619<br>**5: 0.023**<br>6: 0.056<br>**7: 0.047**		
**PD15: CXCL1 Hippocampus**	1: Sex<br>2: Genotype<br>3: MS<br>4: Sex x (genotype)<br>5: Sex x (MS)<br>6: Genotype x MS<br>7: Sex x (Genotype) x (MS)	1: F(1,32)=1.823<br>2: F(1,32)=0.222<br>3: F(1,32)=0.815<br>4: F(1,32)=5.697<br>5: F(1,32)=1.116<br>6: F(1,32)=0.358<br>7: F(1,32)=0.358	1: 0.186<br>2: 0.640<br>3: 0.373<br>**4: 0.023**<br>5: 0.298<br>6: 0.553<br>7: 0.553	-	
**PD15: TNF-α Hippocampus**	1: Sex<br>2: Genotype<br>3: MS<br>4: Sex x (genotype)<br>5: Sex x (MS)<br>6: Genotype x MS<br>7: Sex x (Genotype) x (MS)	1: F(1,32)=4.890<br>2: F(1,32)=2.138<br>3: F(1,32)=0.007<br>4: F(1,32)=1.623<br>5: F(1,32)=2.741<br>6: F(1,32)=0.036<br>7: F(1,32)=2.600	**1: 0.034**<br>2: 0.153<br>3: 0.931<br>4: 0.211<br>5: 0.107<br>6: 0.849<br>7: 0.116	-	

Supplementary Table 2.1: 3-way ANOVA analysis for figure 2.

Experiment	3-way ANOVA			Significant Tukey's multiple comparisons test	
	Factor	F (DFn, DFd)	P Value	Comparisons	P Value
**4 months: Open Field Test**	1: Sex<br>2: Genotype<br>3: MS<br>4: Sex x (genotype)<br>5: Sex x (MS)<br>6: Genotype x MS<br>7: Sex x (Genotype) x (MS)	1: F(1,63)=4.783<br>2: F(1,63)=1.324<br>3: F(1,63)=2.393<br>4: F(1,63)=2.090<br>5: F(1,63)=1.976<br>6: F(1,63)=1.770<br>7: F(1,63)=1.063	**1: 0.032**<br>2: 0.254<br>3: 0.126<br>4: 0.153<br>5: 0.164<br>6: 0.188<br>7: 0.306	WT F Non-MS vs. WT M MS	0.044
				WT F Non-MS vs. 5Xfad M Non-MS	0.031
**4 months: Forced Swim Test**	1: Sex<br>2: Genotype<br>3: MS<br>4: Sex x (genotype)<br>5: Sex x (MS)<br>6: Genotype x MS<br>7: Sex x (Genotype) x (MS)	1: F(1,58)=1.095<br>2: F(1,58)=10.97<br>3: F(1,58)=28.97<br>4: F(1,58)=2.537<br>5: F(1,58)=0.010<br>6: F(1,58)=0.959<br>7: F(1,58)=2.827	1: 0.299<br>**2: 0.001**<br>**3: <0.0001**<br>4: 0.116<br>5: 0.920<br>6: 0.331<br>7: 0.098	WT F MS vs. 5xFAD F Non-MS	0.0002
				WT F MS vs. WT M Non-MS	0.004
				WT F MS vs. 5xFAD M Non-MS	0.003
				5xFAD F vs. WT M MS	0.0002
				WT M Non-MS vs. WT M MS	0.007
				WT M MS vs. 5xFAD M Non-MS	0.004
**4 months: Novel Object Recognition Memory Test**	1: Sex<br>2: Genotype<br>3: MS<br>4: Sex x (genotype)<br>5: Sex x (MS)<br>6: Genotype x MS<br>7: Sex x (Genotype) x (MS)	1: F(1,39)=3.980<br>2: F(1,39)=8.221<br>3: F(1,39)=29.44<br>4: F(1,39)=1.990<br>5: F(1,39)=0.002<br>6: F(1,39)=0.044<br>7: F(1,39)=$7.6*10^{-8}$	1: 0.053<br>**2: 0.006**<br>**3: <0.0001**<br>4: 0.166<br>5: 0.961<br>6: 0.834<br>7: 0.999	WT F Non-MS vs. 5xFAD F MS	0.027
				WT F Non-MS vs. 5xFAD M MS	<0.0001
				5xFAD F Non-MS vs. 5xFAD M MS	0.0006
				WT M Non-MS vs. 5xFAD M MS	0.0004
**4 months: HP gene expression *Bdnf***	1: Sex<br>2: Genotype<br>3: MS<br>4: Sex x (genotype)<br>5: Sex x (MS)<br>6: Genotype x MS<br>7: Sex x (Genotype) x (MS)	1: F(1,37)=1.200<br>2: F(1,37)=0.384<br>3: F(1,37)=18.10<br>4: F(1,37)=0.491<br>5: F(1,37)=4.147<br>6: F(1,37)=0.197<br>7: F(1,37)=0.081	1: 0.280<br>2: 0.539<br>**3: 0.0001**<br>4: 0.487<br>**5: 0.048**<br>6: 0.659<br>7: 0.776	WT F MS vs. WT M Non-MS	0.033
				WT M Non-MS vs. 5xFAD M MS	0.004
**4 months: HP gene expression *Arc***	1: Sex<br>2: Genotype<br>3: MS<br>4: Sex x (genotype)<br>5: Sex x (MS)<br>6: Genotype x MS<br>7: Sex x (Genotype) x (MS)	1: F(1,40)=0.008<br>2: F(1,40)=1.063<br>3: F(1,40)=0.483<br>4: F(1,40)=0.186<br>5: F(1,40)=1.753<br>6: F(1,40)=0.017<br>7: F(1,40)=10.32	1: 0.927<br>2: 0.308<br>3: 0.490<br>4: 0.668<br>5: 0.193<br>6: 0.894<br>**7: 0.002**	-	

7 **Supplementary Table 3.1:** 3-way ANOVA analysis for figure 3.

Experiment	3-way ANOVA			Significant Tukey's multiple comparisons test	
	Factor	F (DFn, DFd)	P Value	Comparisons	P Value
**4 months: Iba1+ area (%) Prefrontal Cortex**	1: Sex<br>2: Genotype<br>3: MS<br>4: Sex x (genotype)<br>5: Sex x (MS)<br>6: Genotype x MS<br>7: Sex x (Genotype) x (MS)	1: F(1,43)=3.143<br>2: F(1,43)=81.78<br>3: F(1,43)=4.368<br>4: F(1,43)=5.522<br>5: F(1,43)=3.873<br>6: F(1,43)=0.244<br>7: F(1,43)=6.031	1: 0.083<br>**2: <0.0001**<br>**3: 0.042**<br>**4: 0.023**<br>5: 0.055<br>6: 0.623<br>**7: 0.018**	WT F Non-MS vs. 5xFAD F Non-MS	0.0067
				WT F Non-MS vs. 5xFAD F MS	<0.0001
				WT F Non-MS vs. 5xFAD M Non-MS	0.003
				WT F Non-MS vs. 5xFAD M MS	0.035
				WT F MS vs. 5xFAD Non-MS F	0.012
				WT F MS vs. 5xFAD F MS	<0.0001
				WT F MS vs. 5xFAD M Non-MS	0.005
				5xFAD F Non-MS vs. 5xFAD F MS	0.027
				5xFAD F Non-MS vs. WT M Non-MS	0.002
				5xFAD F MS vs. WT M Non-MS	<0.0001
				5xFAD F MS vs. WT M MS	<0.0001
				5xFAD F MS vs. 5xFAD M Non-MS	0.029
				5xFAD F MS vs. 5xFAD M MS	0.002
				WT M Non-MS vs. 5xFAD M Non-MS	0.001
				WT M Non-MS vs. 5xFAD M MS	0.017
				WT M MS vs. 5xFAD M Non-MS	0.034

**4 months: CD68+ area related to Iba1+ area (%) Prefrontal Cortex**	1: Sex<br>2: Genotype<br>3: MS<br>4: Sex x (genotype)<br>5: Sex x (MS)<br>6: Genotype x MS<br>7: Sex x (Genotype) x (MS)	1: F(1,31)=4.591<br>2: F(1,31)=31.58<br>3: F(1,31)=0.230<br>4: F(1,31)=0.879<br>5: F(1,31)=1.467<br>6: F(1,31)=4.372<br>7: F(1,31)=2.890	**1: 0.040**<br>**2: <0.0001**<br>3: 0.634<br>4: 0.355<br>5: 0.235<br>**6: 0.044**<br>7: 0.099	WT F Non-MS vs. 5xFAD F Non-MS	0.025
				WT F Non-MS vs. 5xFAD F MS	0.004
				WT F Non-MS vs. 5xFAD M Non-MS	0.031
				5xFAD F Non-MS vs. WT M Non-MS	0.002
				5xFAD F MS vs. WT M Non-MS	0.0004
				5xFAD F MS vs. WT M MS	0.044
				WT M Non-MS vs. 5xFAD M Non-MS	0.003

Supplementary Table 4.1: 3-way ANOVA analysis for figure 4.

Experiment	3-way ANOVA			Significant Tukey's multiple comparisons test	
	Factor	F (DFn, DFd)	P Value	Comparisons	P Value
**4 months: Inflammatory Monocytes**	1: Sex<br>2: Genotype<br>3: MS<br>4: Sex x (genotype)<br>5: Sex x (MS)<br>6: Genotype x MS<br>7: Sex x (Genotype) x (MS)	1: F(1,60)=9.208<br>2: F(1,60)=0.376<br>3: F(1,60)=22.07<br>4: F(1,60)=3.128<br>5: F(1,60)=1.091<br>6: F(1,60)=0.069<br>7: F(1,60)=1.481	**1: 0.003**<br>2: 0.541<br>**3: <0.0001**<br>4: 0.082<br>5: 0.300<br>6: 0.792<br>7: 0.228	WT F Non-MS vs. 5xFAD M MS	0.001
				5xFAD F Non-MS vs. WT M MS	0.043
				5xFAD F Non-MS vs. 5xFAD M MS	0.001
				5xFAD F MS vs. 5xFAD M MS	0.041
				WT M Non-MS vs. 5xFAD M MS	0.016
**4 months: Cytotoxic T Cells**	1: Sex<br>2: Genotype<br>3: MS<br>4: Sex x (genotype)<br>5: Sex x (MS)<br>6: Genotype x MS<br>7: Sex x (Genotype) x (MS)	1: F(1,60)=9.820<br>2: F(1,60)=3.152<br>3: F(1,60)=20.54<br>4: F(1,60)=0.189<br>5: F(1,60)=0.956<br>6: F(1,60)=2.551<br>7: F(1,60)=1.176	**1: 0.002**<br>2: 0.080<br>**3: <0.0001**<br>4: 0.665<br>5: 0.332<br>6: 0.115<br>7: 0.282	WT F Non-MS vs. 5xFAD M MS	0.046
				WT F MS vs. 5xFAD F Non-MS	0.002
				5xFAD F Non-MS vs. 5xFAD F MS	0.028
				5xFAD F Non-MS vs. WT M Non-MS	0.005
				5xFAD F Non-MS vs. WT M MS	0.0007
				5xFAD F Non-MS vs. 5xFAD M MS	0.001
**4 months: Dendritic Cells**	1: Sex<br>2: Genotype<br>3: MS<br>4: Sex x (genotype)<br>5: Sex x (MS)<br>6: Genotype x MS<br>7: Sex x (Genotype) x (MS)	1: F(1,60)=4.610<br>2: F(1,60)=8.868<br>3: F(1,60)=4.649<br>4: F(1,60)=0.122<br>5: F(1,60)=0.022<br>6: F(1,60)=0.015<br>7: F(1,60)=2.370	**1: 0.035**<br>**2: 0.004**<br>**3: 0.035**<br>4: 0.728<br>5: 0.880<br>6: 0.902<br>7: 0.128	-	
**4 months: Eosinophils**	1: Sex<br>2: Genotype<br>3: MS<br>4: Sex x (genotype)<br>5: Sex x (MS)<br>6: Genotype x MS<br>7: Sex x (Genotype) x (MS)	1: F(1,60)=5.729<br>2: F(1,60)=0.771<br>3: F(1,60)=7.144<br>4: F(1,60)=1.684<br>5: F(1,60)=0.672<br>6: F(1,60)=1.134<br>7: F(1,60)=0.020	**1: 0.019**<br>2: 0.383<br>**3: 0.009**<br>4: 0.199<br>5: 0.415<br>6: 0.291<br>7: 0.887	-	
**4 months: Neutrophils**	1: Sex<br>2: Genotype<br>3: MS<br>4: Sex x (genotype)<br>5: Sex x (MS)<br>6: Genotype x MS	1: F(1,60)=14.56<br>2: F(1,60)=1.769<br>3: F(1,60)=3.980<br>4: F(1,60)=0.005<br>5: F(1,60)=0.021<br>6: F(1,60)=1.730	**1: 0.0003**<br>2: 0.188<br>3: 0.050<br>4: 0.942<br>5: 0.883<br>6: 0.193	WT F Non-MS vs. 5xFAD M MS	0.005
				5xFAD F Non-MS vs. 5xFAD M MS	0.042

	7: Sex x (Genotype) x (MS)	7: F(1,60)=2.349	7: 0.130		
**4 months: Activated T helper**	1: Sex<br>2: Genotype<br>3: MS<br>4: Sex x (genotype)<br>5: Sex x (MS)<br>6: Genotype x MS<br>7: Sex x (Genotype) x (MS)	1: F(1,60)=2.886<br>2: F(1,60)=14.17<br>3: F(1,60)=5.226<br>4: F(1,60)=$8.1*10^{-6}$<br>5: F(1,60)=0.649<br>6: F(1,60)=2.519<br>7: F(1,60)=1.053	1: 0.094<br>**2: 0.0004**<br>**3: 0.025**<br>4: 0.997<br>5: 0.423<br>6: 0.117<br>7: 0.309	WT F MS vs. 5xFAD M Non-MS<br><br>WT M MS vs. 5xFAD M Non-MS	0.031<br><br>0.026
**4 months: Activated T cytotoxic**	1: Sex<br>2: Genotype<br>3: MS<br>4: Sex x (genotype)<br>5: Sex x (MS)<br>6: Genotype x MS<br>7: Sex x (Genotype) x (MS)	1: F(1,60)=0.025<br>2: F(1,60)=2.263<br>3: F(1,60)=1.268<br>4: F(1,60)=0.257<br>5: F(1,60)=0.109<br>6: F(1,60)=4.381<br>7: F(1,60)=3.808	1: 0.873<br>2: 0.137<br>3: 0.264<br>4: 0.613<br>5: 0.741<br>**6: 0.040**<br>7: 0.055	-	

Supplementary Table 5.1: 3-way ANOVA analysis for figure Supplementary 1.

Experiment	3-way ANOVA			Significant Tukey's multiple comparisons test	
	**Factor**	**F (DFn, DFd)**	**P Value**	**Comparisons**	**P Value**
**4 months: Iba1 Dentate Gyrus**	1: Sex<br>2: Genotype<br>3: MS<br>4: Sex x (genotype)<br>5: Sex x (MS)<br>6: Genotype x MS<br>7: Sex x (Genotype) x (MS)	1: F(1,46)=3.326<br>2: F(1,46)=20.29<br>3: F(1,46)=0.021<br>4: F(1,46)=1.075<br>5: F(1,46)=0.471<br>6: F(1,46)=0.198<br>7: F(1,46)=1.211	1: 0.074<br>**2: <0.0001**<br>3: 0.883<br>4: 0.305<br>5: 0.495<br>6: 0.658<br>7: 0.276	WT F Non-MS vs. 5xFAD F MS	0.049
				WT F MS vs. 5xFAD F MS	0.040
				5xFAD F MS vs. WT M Non-MS	0.009
**4 months: Iba1 CA1**	1: Sex<br>2: Genotype<br>3: MS<br>4: Sex x (genotype)<br>5: Sex x (MS)<br>6: Genotype x MS<br>7: Sex x (Genotype) x (MS)	1: F(1,46)=3.621<br>2: F(1,46)=21.84<br>3: F(1,46)=0.394<br>4: F(1,46)=1.042<br>5: F(1,46)=0.147<br>6: F(1,46)=0.232<br>7: F(1,46)=1.502	1: 0.063<br>**2: <0.0001**<br>3: 0.533<br>4: 0.312<br>5: 0.702<br>6: 0.632<br>7: 0.226	WT F Non-MS vs. 5xFAD F MS	0.032
				WT F MS vs. 5xFAD F MS	0.007
				5xFAD F MS vs. WT M Non-MS	0.002
				5xFAD F MS vs. WT M MS	0.027
**4 months: Iba1 CA3**	1: Sex<br>2: Genotype<br>3: MS<br>4: Sex x (genotype)<br>5: Sex x (MS)<br>6: Genotype x MS<br>7: Sex x (Genotype) x (MS)	1: F(1,45)=1.348<br>2: F(1,45)=7.708<br>3: F(1,45)=0.406<br>4: F(1,45)=0.887<br>5: F(1,45)=0.001<br>6: F(1,45)=0.010<br>7: F(1,45)=1.024	1: 0.251<br>**2: 0.008**<br>3: 0.526<br>4: 0.351<br>5: 0.971<br>6: 0.979<br>7: 0.317	-	
**4 months: Iba1 Amygdala**	1: Sex<br>2: Genotype<br>3: MS<br>4: Sex x (genotype)<br>5: Sex x (MS)<br>6: Genotype x MS<br>7: Sex x (Genotype) x (MS)	1: F(1,45)=0.124<br>2: F(1,45)=20.74<br>3: F(1,45)=0.046<br>4: F(1,45)=0.500<br>5: F(1,45)=0.500<br>6: F(1,45)=0.032<br>7: F(1,45)=2.276	1: 0.725<br>**2: <0.0001**<br>3: 0.829<br>4: 0.482<br>5: 0.482<br>6: 0.857<br>7: 0.138	WT F MS vs. 5xFAD F MS	0.019
				WT F MS vs. 5xFAD M Non-MS	0.047
				5xFAD F MS vs. WT M Non-MS	0.041
